# The αC-β4 loop controls the allosteric cooperativity between nucleotide and substrate in the catalytic subunit of protein kinase A

**DOI:** 10.1101/2023.09.12.557419

**Published:** 2023-09-15

**Authors:** Cristina Olivieri, Yingjie Wang, Caitlin Walker, Manu V. Subrahmanian, Kim N. Ha, David A. Bernlohr, Jiali Gao, Carlo Camilloni, Michele Vendruscolo, Susan S. Taylor, Gianluigi Veglia

**Affiliations:** 1 Department of Biochemistry, Molecular Biology, and Biophysics, University of Minnesota, MN 55455, USA; 2 Department of Chemistry and Supercomputing Institute, University of Minnesota, MN 55455, USA; 3 Departmenf of Chemistry and Biochemistry, St. Catherine University, MN 55105, USA; 4 Department of Chemistry, University of Cambridge, Cambridge CB2 1EW, UK.; 5 Department of Pharmacology, University of California at San Diego, CA 92093, USA.; 6 Department of Chemistry and Biochemistry, University of California at San Diego, CA 92093, USA.

**Keywords:** Protein Kinases, cAMP-dependent protein kinase A, allosteric mutations, binding cooperativity

## Abstract

Allosteric cooperativity between ATP and substrates is a prominent characteristic of the cAMP-dependent catalytic (C) subunit of protein kinase A (PKA). Not only this long-range synergistic action is involved in substrate recognition and fidelity, but it is likely to regulate PKA association with regulatory subunits and other binding partners. To date, a complete understanding of the molecular determinants for this intramolecular mechanism is still lacking.

Here, we used an integrated NMR-restrained molecular dynamics simulations and a Markov Model to characterize the free energy landscape and conformational transitions of the catalytic subunit of protein kinase A (PKA-C). We found that the apo-enzyme populates a broad free energy basin featuring a conformational ensemble of the active state of PKA-C (ground state) and other basins with lower populations (excited states). The first excited state corresponds to a previously characterized inactive state of PKA-C with the αC helix swinging outward. The second excited state displays a disrupted hydrophobic packing around the regulatory (R) spine, with a flipped configuration of the F100 and F102 residues at the tip of the αC-β4 loop. To experimentally validate the second excited state, we mutated F100 into alanine and used NMR spectroscopy to characterize the binding thermodynamics and structural response of ATP and a prototypical peptide substrate. While the activity of PKA-C^F100A^ toward a prototypical peptide substrate is unaltered and the enzyme retains its affinity for ATP and substrate, this mutation rearranges the αC-β4 loop conformation interrupting the allosteric coupling between nucleotide and substrate. The highly conserved αC-β4 loop emerges as a pivotal element able to modulate the synergistic binding between nucleotide and substrate and may affect PKA signalosome. These results may explain how insertion mutations within this motif affect drug sensitivity in other homologous kinases.

## INTRODUCTION

Eukaryotic protein kinases (EPKs) are plastic enzymes of paramount importance to signaling processes, catalyzing phosphoryl transfer reactions, or acting as scaffolds for other enzymes and/or binding partners ^1^. Of all kinases, the catalytic subunit of PKA (PKA-C) was the first to be structurally characterized by X-ray crystallography.^2,3^ In its inhibited state, PKA-C forms a heterotetrametric complex comprising two catalytic (C) and two regulatory (R) subunits.^4^ The canonical activation mechanism of PKA involves binding two cAMP molecules and disassembling the holoenzyme, which unleashes active PKA-C monomers that target signaling partners.^5^ In 1997, however, Scott and coworkers suggested that the holoenzyme does not disassemble under physiological conditions; rather, the intact holo-enzyme forms *signaling islands* localized by A-kinase anchoring proteins (AKAPs) in the proximity of substrates.^6^

**Figure 1.**
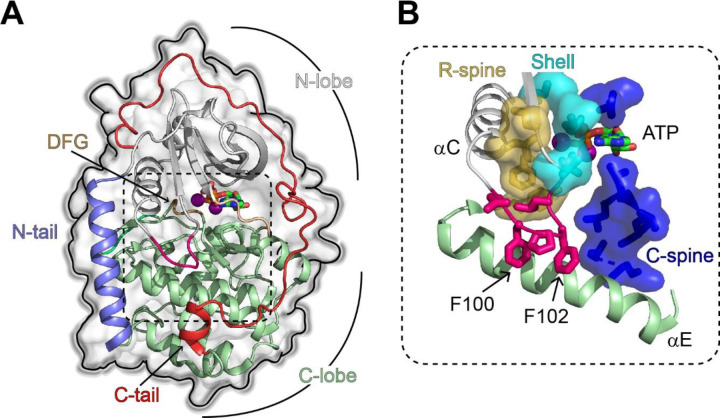
Structural and catalytic motifs of PKA-C. (**A**) Surface representation of the X-ray structure of PKA-C bound to the endogenous inhibitor, PKI (PDB: 4WB5). (**B**) Hydrophobic organization of the PKA-C core, with the R-spine (gold), C-spine (blue), shell residues (cyan), and the αC-β4 loop (hot pink) that locks into αE helix.

X-ray crystallography studies revealed that PKA-C is a bilobal enzyme, with a dynamic N-terminal lobe comprising β-sheets and the αC helix and a more rigid C-terminal lobe, with mostly α-helices (Figure 1).^2,3,7^ The N-lobe harbors the nucleotide-binding site, whereas the substrate binding cleft lays at the interface between the N-lobe and C-lobe. The three-dimensional structure of the enzyme features a highly conserved hydrophobic core decorated by catalytically important motifs, *i.e.*, the Gly-rich loop, DFG-loop, activation loop, positioning loop, and magnesium and peptide positioning loops.^8^ In the catalytically active state, these motifs are all poised for phosphoryl transfer. However, the conformation of these motifs is necessary but not sufficient to define an active kinase. More recent studies revealed a critical role of the hydrophobic core, which is crossed by a catalytic (C) spine, a regulatory (R) spine, and shell residues (Figure 1).^9,10^ The C spine comprises an array of hydrophobic residues and is assembled upon binding ATP, whereas the R spine is engaged when the activation loop is phosphorylated, which contributes to the positioning of the αC-helix.^11^

A distinct property of PKA-C is the binding cooperativity between ATP and substrate.^12^ During the catalytic cycle, the kinase recognizes and binds substrates with positive binding cooperativity between ATP and unphosphorylated substrates, whereas a negative binding cooperativity between ADP and phosphorylated substrate characterizes the exit complex.^13^ The biological importance of binding cooperativity has been emphasized by our recent studies on disease-driven mutations of PKA-C,^14–16^ which all feature disrupted cooperativity between nucleotides and protein kinase inhibitor (PKI) or typical substrates.^14–16^ Since the recognition sequence of the substrate is highly homologous to the regulatory subunits,^4^ a loss of cooperativity of binding may affect not only substrate binding fidelity but also the regulation by the R subunit. However, the molecular determinants for the binding cooperativity between ATP and substrate and its role in PKA signalosome remain elusive to date.

Here, we combined NMR-restrained replica-averaged metadynamics (RAM)^17,18^ and Markov State Model (MSM)^19^ to define the conformational landscape and the corresponding dynamics of PKA-C. We found that both the apo kinase and the nucleotide-bound kinase occupy three distinct basins: (*i*) a most populated ground state with constitutively active conformations competent for catalysis, (*ii*) a first high free energy basin representative of typical inactive states with a dislodged configuration of the αC helix, and (*iii*) a second high free energy basin with a disrupted hydrophobic array of residues at the core of the enzyme. Notably, the equilibrium between the most populated ground state and the other low-populated states agrees with previous NMR relaxation dispersion and CEST measurements.^20^ To compound the existence of the second inactive basin, we mutated F100 at the tip of the αC-β4 loop into Ala (PKA-C^F100A^) and characterized its binding thermodynamics and ligand binding response by isothermal titration calorimetry and NMR spectroscopy. We found that PKA-C^F100A^ phosphorylates canonical peptides and preserves the binding affinity for both nucleotide and substrate. However, PKA-C^F100A^ lacks binding cooperativity between nucleotide and substrate. NMR revealed that perturbing the hydrophobic packing around the αC-β4 loop interrupts the allosteric communication between the two lobes of the enzyme. These results further support the pivotal role of the αC-β4 loop in kinases function and may explain why single-site mutations or insertion mutations in the homologous kinase that stabilize this motif result in oncogenes and confer different drug sensitivity.^21^

## RESULTS

### The free energy landscape of PKA-C charted by NMR-restrained replica-averaged metadynamics (RAM) simulations.

**Figure 2.**
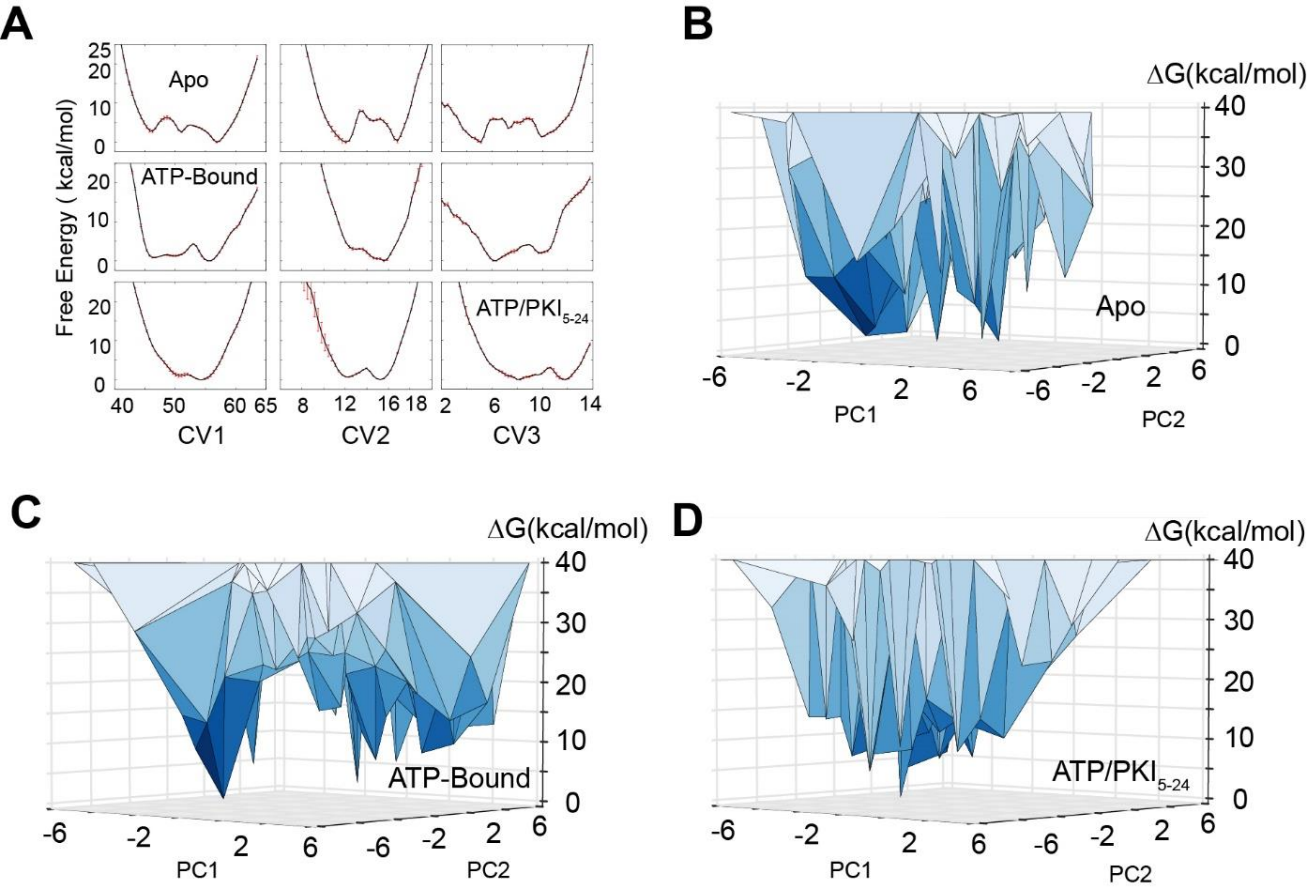
Free energy landscape (FEL) of PKA-C in various ligated forms obtained from replica-averaged metadynamics (RAM) simulations. (**A**) Convergence of the bias deposition along the first three collective variables (CVs). The free energy (expressed in kcal/mol) of the different CVs were averaged over the last 100 ns of RAM simulations. The standard deviations are reported as red error bars. (**B-D**) FEL along the first two principal components (PC1 and PC2) of PKA-C in the apo, ATP-bound, and ATP and the model substrate PKI bound forms. PC1 and PC2 are projected from the first three CVs. The vertices represent conformational states. In the apo form, multiple states have comparable free energy with ∆G < 5 kcal/mol, whereas in the binary form, fewer states have ∆G < 5 kcal/mol, whereas for the ternary form only a major ground state is populated.

To characterize the experimentally accessible conformational landscape of PKA-C in the µs-to-ms range, we performed NMR-restrained metadynamics simulations within the RAM framework.^17,18^ We simulated the apo, binary (PKA-C/ATP), and ternary (PKA-C/ATP/PKI_5–24_) forms of PKA-C, using four replicas restrained using backbone chemical shifts (CS) to ensure a close agreement with conformational space explored by the kinase under the conditions used in this work. The enhanced sampling was achieved through bias-exchange metadynamics along different collective variables (CVs) to boost the conformational plasticity of the enzyme (Figure 2 - figure supplement 1).^22^ Back-calculation of the CS using the Sparta+ software^23^ shows that the CS restraints improved the agreement between back-calculated and experimental CS. Specifically, we obtained an overall improvement of ~0.2 ppm on the amide N atom and ~0.1 ppm on the remainder backbone atoms (Figure 2 - figure supplement 2). The bias-exchange metadynamics allowed for each replica to span a broader conformational space relative to classical simulations (Figure 2 - figure supplement 3). The deposition of Gaussian biases required by the metadynamics approach converged after 300 ns, where the fluctuations along the first 3 CVs were less than 1 kcal/mol (Figure 2A, Figure 2 - figure supplement 4). The converged bias revealed that the *apo* kinase accesses multiple minima along each CV, and the conformational heterogeneity is significantly reduced upon ligand binding, especially in the ternary form (Figure 2A ). The full free energy landscape was then reconstructed by sampling an extra 100 ns production phase with reduced biases along each CV. The free energy landscape shows how the population of the conformers is modulated by ligands within the NMR detection limit of sparsely populated states (~ 0.5% or ∆G < 3.2 kcal/mol).

According to these simulations, apo PKA-C populates preferentially a ground state and five readily accessible low-populated excited states (Figure 2B, Figure 2 – supplementary table 1). The nucleotide-bound PKA-C (binary form) features a similar ground state and a broad higher energy basin (Figure 2C, Figure 2 – supplementary table 1). Finally, the ternary complex occupies a narrow dominant ground state (Figure 2D, Figure 2 – supplementary table 1). This free energy landscape obtained from the RAM simulations is consistent with the qualitative picture previously inferred from our NMR spin relaxation experiments,^24^ while providing a detailed structural characterization of the excited states.

### MSM reveals the conformational transitions of PKA-C from ground to high free energy (excited) states.

**Figure 3.**
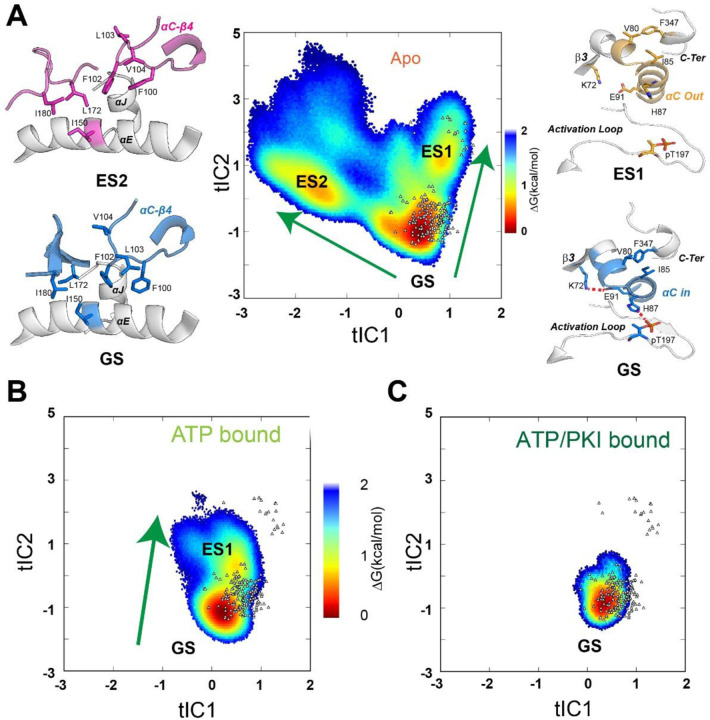
The apo, ATP-bound, and ATP/PKI-bound PKA-C reveal distinct free energy surface (FES) and dynamics, as determined by a Markov State Model (MSM). (**A**) Free energy landscape projected along the first two time-lagged independent components (tICs) of the apo PKA-C, the projections of known crystal structures, and characteristic features of GS, ES1, and ES2. The transition from GS to ES1 highlights the changes around the αB-αC loop, where the salt bridges between K72-E91 and H87-T197 and the PIF pocket (V80-I85-F347) are all disrupted. The transition from GS to ES2 highlights the rearrangement around the αC-β4 loop, with distinct local hydrophobic packing. (**B** and **C**) FES projected along the first two tICs for the ATP-bound PKA-C (**B**), ATP/PKI bound PKA-C (**C**), and the projections of known crystal structures.

To explore the conformational transitions of the kinase upon ligand binding, we performed additional unbiased sampling to build a Markov State Model (MSM). MSMs are commonly used to describe the dynamic transitions of macromolecules in terms of (1) the probabilities of occupation of a specific set of states and (2) the transition probabilities of moving between these states. In practice, a MSM is typically created by combining thousands of short unbiased simulations. ^25,26^ Following this strategy, we performed several short simulations (10 – 20 ns) using thousands of the low free energy conformations (∆G < 3.2 kcal/mol) chosen randomly from the three forms of PKA-C as starting structures. The conformational ensembles were clustered into microstates and seeded to start a second round of adaptive sampling (see Methods). The iterative process was repeated three times to assure convergence and yielded a total of 100 μs trajectories for both the apo and binary forms, whereas for the less dynamic ternary complex, we collected trajectories of 60 μs. Once we reached a sufficient sampling, we built a MSM including L95, V104, L106, M118, M120, Y164, and F185 to investigate the dynamic transitions of the hydrophobic R spine and shell residues (Figure 3 – figure supplement 1). These residues are ideal reporters of the dynamic processes governing the activation/deactivation of the kinase.^27^ To compare the free energy landscape of different complexes, we first projected the conformational ensembles of three forms and existing crystal structures along the first two time-lagged independent components (tICs) of the *apo* form, which were obtained by a time-lagged independent component analysis (tICA) analysis (see Methods). These tICs represent the directions of the slowest motion of the kinase and visualize the conformational transitions of V104, L95, and F185 (Figure 3 – figure supplement 1). Apo PKA-C accesses three major basins. The broadest basin represents the ground state (GS) and represents the conformations of the kinase captured by essentially all crystal structures (Figure 3A). Additionally, there are two distinct excited states: the first excited state (ES1) features a disrupted hydrophobic packing of L95, V104, and F185. This conformation features an inactive state with an orientation of the αC-helix typical of the inhibited states as found for the PKA-C bound to regulatory subunits RIα and RIIβ. The second excited state (ES2), to our knowledge, was never captured in crystal structures. The ES2 state displays a flipped configuration of the V104 side chain and a rearrangement of the αC-β4 loop, with the dihedral angles of F100 and F102 adopting a gauche^+^ configuration. This orientation of the F100 and F102 aromatic rings breaks the hydrophobic packing of the αC-β4 loop with the C-lobe, causing steric contacts between F100 and V104 (Figure 3A ). In contrast, the active GS ensemble features a *trans* configuration of the F100 and F102 side chains that stabilizes the hydrophobic interactions with αE- and αJ-helices in the C-lobe (Figure 3A ). Upon binding ATP, the conformational space span by the kinase becomes narrower, and the conformers populate mostly the GS, with a small fraction in the ES1 state (Figure 3B). This is consistent with the role of the nucleotide as an allosteric effector, enhancing the affinity of the enzyme for the substrate. In the ternary form (ATP and PKI-bound), PKA-C populates only the GS consistent with the competent conformation observed in the first ternary complex structure. In this case, the αC-β4 loop of the enzyme is locked in a well-defined configuration, as shown in Figure 3A.

**Figure 4.**
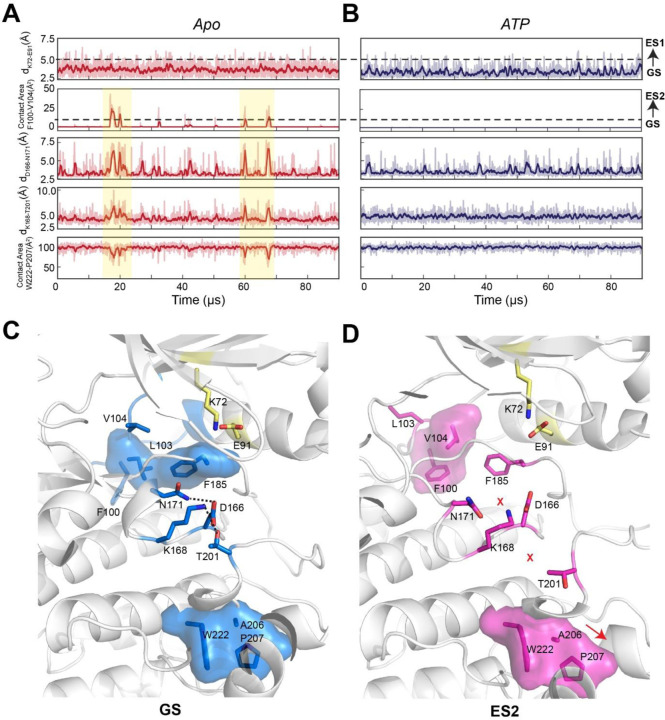
Conformational transition between GS and ES1, ES2 along the kinetic Monte Carlo trajectory in apo (A) and ATP-bound (B) forms. **(A, B)** The transition from GS to ES1, revealed as breaking of the K72-E91 salt bridge, is frequently found in both forms, whereas the transition to ES2, revealed as the contact between F100 and V104, only occurs in the apo form and in concert with allosteric changes between D166-N171, K168-T201, and W222-A206-P207. The darker colors in (A) and (B) highlight moving averages over every 10 frames. (C) GS conformation reveals the assembly of key catalytic features across the core region. **(D)** ES2 conformation revealed disruption of key structure motifs across the core region, indicative of inactivation.

In the apo form, the αC-β4 loop is quite dynamic due to transient hydrophobic interactions between F100 and V104 as well as W222 and the APE motif (A206 and P207) (Figure 4A). The binding of both nucleotide and PKI increases the rigidity of residues near F100 and V104 such as V103 and F185 (Figure 4A). In addition, several electrostatic interactions essential for catalysis (D166-N171, K168-T201, and Y204-E230), which are transient in the apo PKA-C, become more persistent (Figure 4B).

The MSM makes it possible to use kinetic Monte Carlo sampling to characterize the slow transition between different states.^28^ In the apo form, the GS features F100 and F102 in *trans*, a configuration that stabilizes the interactions with the αE and αJ helices and, together with the nucleotide, locks the αC-β4 loop to elicit an active kinase conformation (Figure 3A). The GS to ES1 transition features the disruption of the K72-E91 salt bridge, whereas the GS to ES2 transition involves a 120° flip of the F100 aromatic group that interacts with V104, a conformation found only in the uncommitted apo enzyme (Figure 3A). The GS to ES2 transition involves a concerted disruption of the D166-N171 and K168-T201 electrostatic interactions, essential for catalysis. Also, this event destabilizes the packing between W222 and the APE motif (A206 and P207) required for substrate recognition (Figure 3C). All these conformational transitions suggest that ES1 and ES2 represent destabilized states of the kinase.

### Direct correspondence between the conformationally excited states identified by MD simulations and NMR.

**Figure 5.**
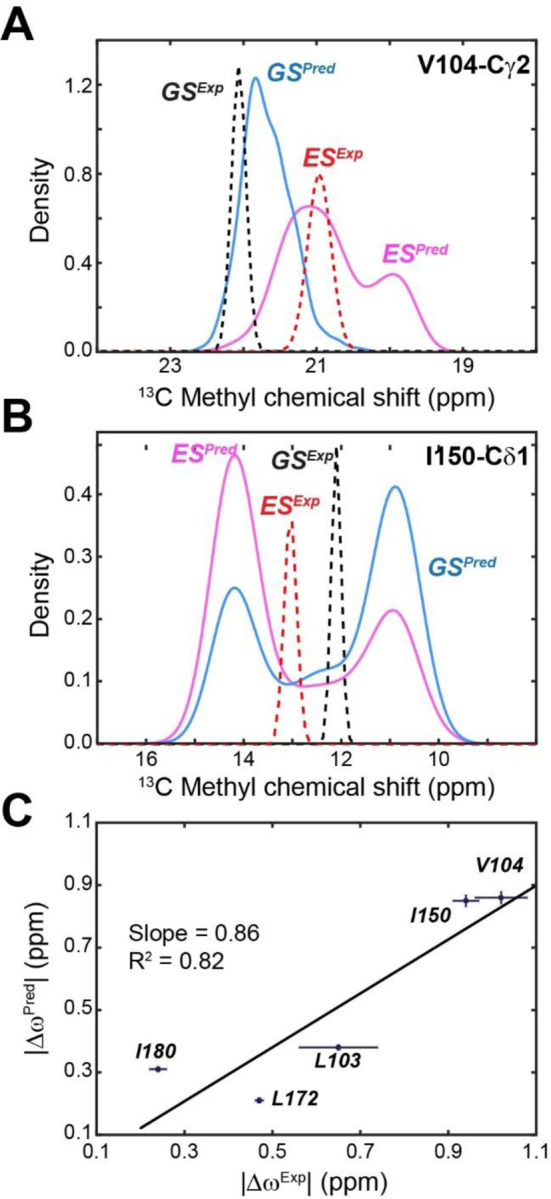
Transition from GS to ES2 shown in the apo PKA-C recapitulated the structural changes near the αC- β4 loop probed by NMR experiments. (**A** and **B**) Distribution of predicted ^13^C CS of selected methyl groups in ES (magenta) and GS (blue) of the apo PKA-C for Val104-Cγ1 (**A**) and Ile150-Cδ1 (**B**). The experimental CS is shown in dotted lines for GS (black) and ES (red). (**C**). Correlation of the predicted chemical shift differences | ∆ω^Pred^| and the experimental result | ∆ω^Exp^| for a set of hydrophobic residues near the αC-β4 loop. The fitted linear correlation has a slope of 0.86 and R^2^ of 0.82.

NMR relaxation dispersion and CEST experiments performed on the apo PKA-C revealed the presence of conformationally excited states for several residues embedded into the hydrophobic core of the enzyme.^20^ Based on the free energy landscape and the conformational transitions in the core of the kinase, we reasoned that the inactive states identified by the simulations would correspond to a disruption of the hydrophobic packing near the αC-β4 loop. To test this hypothesis, we calculated the values of the change in CS (∆ω) for the methyl groups of L103, V104, I150, L172, and I180 from the MD simulations and compared them with those obtained from the fitting of the CPMG dispersion curves and CEST profiles.^20^ We first sampled more than 500 snapshots as representative structures of the ES2 and GS states (Figure 3 – figure supplement 2), and computed the distribution of the methyl ^13^C chemical shifts on these sites using ShiftX2.^29^ These calculations yield ∆ω values of 0.87 ± 0.02 and 0.85 ± 0.02 ppm between ES2 and GS for Val104 and Ile150, respectively. These values are in good agreement with the experimental ∆ω values (1.10 ± 0.06 and 0.94 ± 0.09 ppm) obtained from the fitting of the CPMG dispersion curves (Figure 5A–B). The CS difference obtained for the other three sites also yielded a good agreement, with the same direction of the chemical shift changes found experimentally (Figure 5 – figure supplement 1). Remarkably, the fitting of the calculated vs. experimental ∆ω values gives a slope of 0.86 with R^2^ of 0.82 (Figure 4C) supporting that the excited state observed experimentally may correspond to the calculated ensemble. In addition, MSM estimates a population of ES2 of 6 ± 2%, consistent with the 5 ± 1% reported in NMR experiments.^27^ These results support that the alternate conformations identified by NMR represent inactive states and may correspond to the disruption of the hydrophobic anchor of the αC-β4 loop via F100 and F102.

### The F100A mutation disrupts the allosteric network of the kinase.

**Figure 6.**
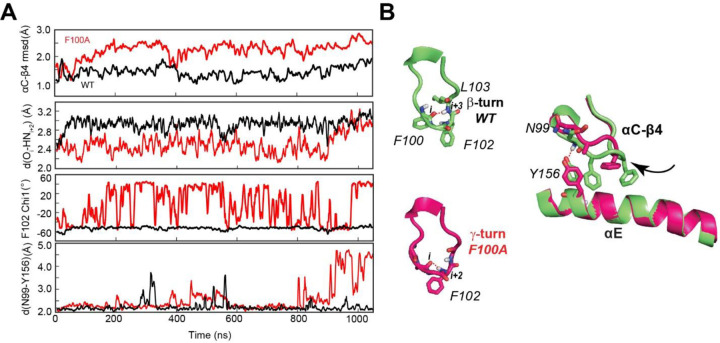
Increased dynamics at the αC-β4 loop upon F100A mutation, perturbing the local hydrophobic packing and its anchoring to the αE helix. (**A**) Time series of the αC-β4 loop, H-bond occurrence for the β– and γ-turns, F102 χ_1_ angle, and N99 and Y156 for WT (black) and F100A (red) in the ATP-bound state. (**B**) Representative structural snapshots showing the formation of the β-turn for the PKA-C^WT^ (green) and γ-turn for the PKA-C^F100A^ mutant.

The above analysis suggests that F100 located at the αC-β4 loop is critical for the GS to ES1 transition path of the kinase. Therefore, we modeled the F100A mutant and analyzed its dynamic trajectories. First, we performed a short equilibration using classical MD simulations starting from the coordinates of the X-ray structure of the ternary complex (PDB ID: 4WB5). During 1 μs of MD simulations, the αC-β4 loop of the F100A mutant undergoes a significant motion as manifested by the increased values of the backbone rmsd and the conformational transition (flipping) of the F102 side chain (Figure 6A). This region in the WT PKA-C adopts a stable β-turn in the WT, with a persistent H-bond between the backbone oxygen of F100 and the amide hydrogen of L103. In the simulation of F100A, H-bond is formed more frequently between A100 and F102, and this region adopts a γ-turn conformation. Such local rearrangement disrupts the hydrogen bond between N99 and Y156 of αE, altering the anchoring of the αC-β4 to the αE helix (Figure 6B).

**Figure 7.**
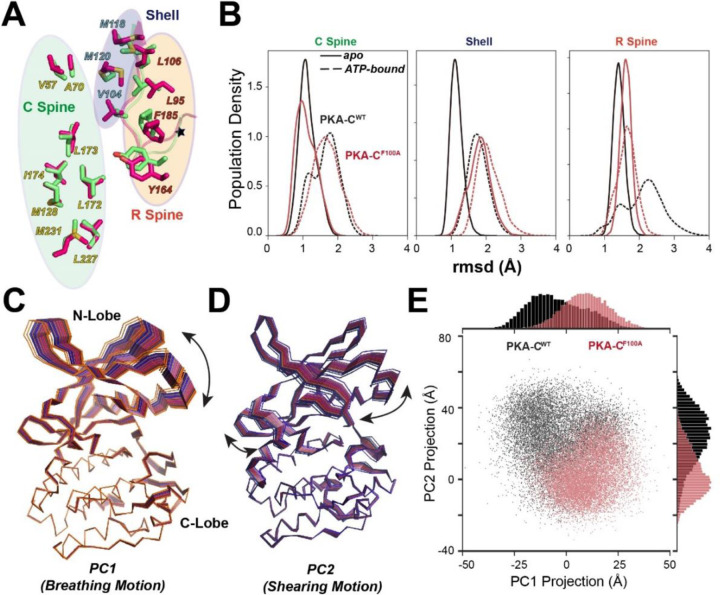
Distinct global structural response to ATP binding in the F100A mutant. **(A)** Structure superposition of the C Spine, R Spine, and Shell residues between WT (lime) and F100A (hot pink), highlighting the differences between Shell and R Spine. **(B)** Change of RMSD upon ATP binding at C Spine, Shell, and R spine, for WT and F100A, respectively. **(C)** Structural illustration of the first principal component (PC1), i.e., the breathing motion of the two lobes. **(D)** Structural illustration of the second principal component (PC2), i.e., the shearing motion of the two lobes. **(E)** Comparison of the 2D projection and distribution along PC1 and PC2 for WT and F100A, highlighting their dramatic differences along both axes.

In the F100A mutant, the local changes caused by the αC-β4 loop structural transitions propagate and alter the response of the nucleotide binding as shown on the rmsd changes for key hydrophobic motifs such as the R- and C- spines and shell residues (Figure 7A). While the nucleotide binding decreases the average RMSD for these hydrophobic motifs for WT PKA-C, it stabilizes only the C-spine and fails to drive the R-spine and shell residues to an intermediate state competent for substrate binding (Figure 7B). The latter can be attributed to the perturbation of hydrophobic packing of L95, L106 of R-spine, and V104 of the shell residues close to the mutation site. Using the lowest principal components, we also analyzed the global dynamic response to ATP binding. Not only does F100A change the breathing mode of the two lobes (PC1), but it also alters the shearing motion (PC2) of the kinase, emphasizing the importance of this allosteric mutation on the internal communication across the hydrophobic core (Figure 7C–E).

**Figure 8.**
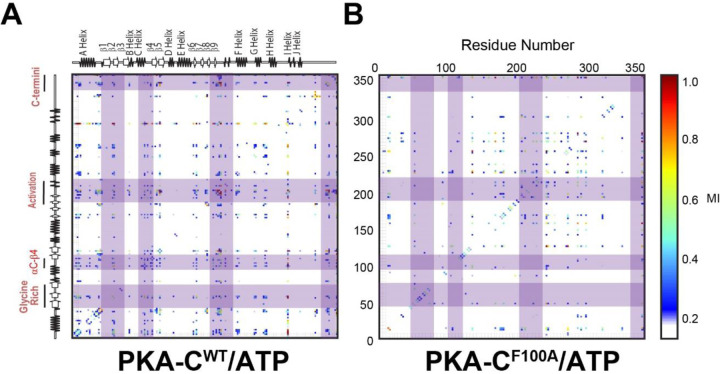
Mutual information of dihedral angles for the (A) WT and (B) F100A upon binding ATP. This analysis reveals the prominent loss of allosteric communication of F100A, especially at multiple key motifs as is highlighted by purple strips.

In fact, these altered motions have global repercussions on the allosteric networks as detected by mutual information for rotameric states (Figure 8). For PKA-C^WT^, there are numerous correlations observable within both lobes of the enzyme, involving the Gly-rich loop, αC-β4 loop, activation loop, and the C-terminal tail These dynamic correlations between the two lobes are the hallmark for the dynamically-committed state. In contrast, the mutual information for F100A mutants shows significantly less correlations in the N-lobe and the correlations within the C-lobe are redistributed.

Taken together, these simulations on F100A suggest that the local perturbation of the αC-β4 loop diminishes the structural connection between the two lobes, disrupting the correlated shearing motion highlighted in other simulation studies. Although the single F100A mutation does not drive the kinase into a completely dysfunctional state (ES2), it is sufficient to abolish the binding cooperativity of the enzyme.

#### The F100A mutation disrupts nucleotide-substrate binding cooperativity in PKA-C.

Based on the results from the MD simulations, we hypothesized that the disruption of the coupling between the N-lobe and C lobe would affect the binding cooperativity between nucleotide (ATPγN) and pseudosubstrate inhibitor (PKI_5–24_) binding. We first evaluated the catalytic efficiency for the wild-type and F100A mutant by carrying out steady-state coupled enzyme assays^30^ using the standard substrate, Kemptide.^31^ F100A showed a slight increase in *K*_*M*_ and *V*_*max*_ compared to PKA-C^WT^, resulting in a reduction of the catalytic efficiency (*k*_*cat*_*/K*_*M*_ = 0.50 ± 0.04 and 0.41 ± 0.08 for PKA-C^WT^ and PKA-C^F100A^, respectively; Supplementary table 2). We then performed isothermal titration calorimetry (ITC)^32^ to obtain ∆*G*, ∆*H*, *-T*∆*S*, *K*_*d*_, and determine the cooperativity coefficient (σ) for ATPγN and PKI_5–24_ binding. We first analyzed the binding of ATPγN to the apo PKA-C^F100A^ and, subsequently, the binding of PKI_5–24_ to the ATPγN-saturated PKA-C^F100A^ (Supplementary Table 3). We found that PKA-C^WT^ and PKAC^F100A^ have similar binding affinities for ATPγN (*K*_*d*_ = 83 ± 8 μM and 73 ± 2 μM, respectively). However, in the apo form F100A showed a 3-fold higher binding affinity for the pseudosubstrate relative to PKA-C^WT^ (*K*_*d*_ = 5 ± 1 μM and 17 ± 2 μM, respectively - Supplementary table 3). Upon saturation with ATPγN, PKA-C^F100A^ displayed a 12-fold reduction in binding affinity for PKI_5–24_, resulting in a σ of ~3, a value significantly lower than the wild-type (σ greater than 100).

#### NMR mapping of nucleotide/pseudosubstrate binding response.

**Figure 9.**
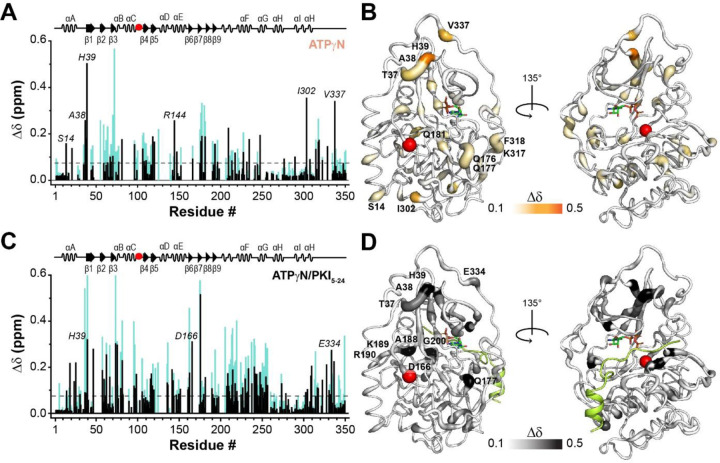
Structural response of PKA-C^F100A^ binding to nucleotide and protein kinase inhibitor. (**A**) Histogram shows the chemical shift perturbation (CSP) of the amide fingerprint for PKA-C^F100A^ (black) in response to ATPγN binding compared to the CSP obtained for the wild-type protein (cyan). The dashed line on the histogram indicates one standard deviation from the average CSP. (**B**) CSPs of PKA-C^F100A^/ATPγN amide resonances mapped onto the structure (PDB: 4WB5). (**C**) CSP of amide fingerprint for PKA-C^F100A^ bound to ATPγN and PKI_5–24_ (black), compared to the CSP of the wild-type protein obtained in the same conditions. (**D**) CSP for the F100A/ATPγN/PKI complex mapped onto the crystal structure (PDB: 4WB5).

To understand the atomic details of the disrupted binding cooperativity for PKA-C^F100A^, we used solution NMR spectroscopy and mapped the amide fingerprints of PKA-C^F100A^ using [^1^H, ^15^N]-WADE-TROSY-HSQC experiments ^20^ upon adding nucleotide (ATPγN) and pseudosubstrate (PKI_5–24_). Specifically, we monitored the ^1^H and ^15^N chemical shift perturbations (CSPs, ∆δ) of the amide fingerprints for the PKA-C^F100A^ saturated with ATPγN and in complex with ATPγN/PKI_5–24_ and we compared them with PKA-C^WT^ (Figure 9 – figure supplement 1). ATPγN binding causes the dramatic broadening of several amide resonances throughout the structure of PKA-C^F100A^, suggesting the presence of an intermediate conformational exchange ^33^. The fingerprints of both PKAC^WT^ and PKA-C^F100A^ show similar CSP profiles upon binding ATPγN (Figure 9 – figure supplement 1B), although the extent of the changes is significantly attenuated for PKA-C^F100A^. The reduction of CSP is apparent for residues in the N-lobe (β2- β3 region), around the mutation (β4), and at the C-terminal tail. A similar pattern is observed upon binding PKI_5–24_ to the ATPγN-saturated PKA-C^F100A^ (Figure 9). A substantial decrease in CSP is observed for the C-lobe near the domains critical for substrate-binding (*i.e.*, αE, αF, and αG).

To determine the global response to ligand binding for WT and F100A, we analyzed the chemical shifts of the amide fingerprints of the two proteins using the COordiNated ChemIcal Shifts bEhavior (CONCISE).^34^ CONCISE describes the global response of the protein fingerprint resonances by providing the probability density (population) of each state along the conformational equilibrium for binding phenomena that follow linear chemical shift trajectories. For PKA-C^WT^, both nucleotide and pseudosubstrate binding shift the overall population of the residues from an open to an intermediate and a fully closed state (Figure 10 – figure supplement 1). A similar trend is observed for PKA-C^F100A^, though the probability densities are broader, indicating that the amide resonances follow a less coordinated response.^34^ Also, the maximum probability density for the closed state shows that the ternary complex of the mutant is slightly more open than the corresponding wild-type (Figure 10 – figure supplement 1). Overall, the shape of the probability distributions of the amide chemical shifts suggests that several residues do not respond in a coordinated manner and that PKI_5–24_ binding shifts the conformation of the kinase toward a partially closed state, which may explain the loss in binding cooperativity as previously observed.^14–16^

**Figure 10 –:**
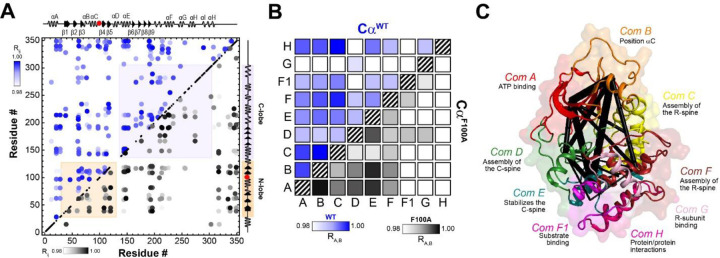
Changes of the intramolecular allosteric network in F100A as mapped by correlated chemical shift changes. **(A)** Comparison of the CHESCA matrices obtained from the analysis of the amide chemical shifts of PKA-C_WT_ (top diagonal, blue) and PKA-C_F100A_ (bottom diagonal, black) in the apo, ADP-bound, ATPγN-bound, and ATPγN/PKI_5–24_-bound states. Only correlations with R_ij_ > 0.98 are reported. The enlarged CHESCA map of F100A is available in Figure 10 – figure supplement 1 while the data for the PKA-C^WT^ matrix are taken from Walker *et al.*^15^. **(B)** Community CHESCA analysis of PKA-C^WT^ (top diagonal, blue) and PKA-C^F100A^ (bottom diagonal, black). Only correlations with R_A,B_ > 0.98 are shown. **(C)** Community CHESCA matrix plotted on its corresponding structures. The size of each node is independent of the number of residues it encompasses while the weight of each line indicates the strength of coupling between the individual communities.

To analyze these coordinated chemical shift changes in detail and define the allosteric network of the kinase upon binding ligands and substrate, we utilized the CHEmical Shift Covariance Analysis (CHESCA),^15,16,20^ a statistical method that identifies correlated responses of residues to a specific perturbation. CHESCA works under the assumption that pairwise correlated chemical shift changes between residues identify possible allosteric networks.^35,36^ We found that coordinated structural rearrangements, as identified by CHESCA, are a strong indication of binding cooperativity in PKA-C.^15,16,20^ Therefore, we compared the CHESCA maps for PKA-C^WT^ and PKAC^F100A^ for four different states: *apo*, ATPγN-, ADP-, and ATPγN/PKI_5–24_-bound. The CHESCA matrix of PKA-C^F100A^ exhibits sparser and more attenuated (i.e., lower value of correlation coefficient) correlations relative to PKA-C^WT^ (Figure 10). Although many inter-lobe correlations are still present for the mutant, several correlations in specific structural domains such as the αG-, αH-, and αI-helices are absent or attenuated. For instance, the F100A mutation does not display correlations between the αA-helix and the C-terminal tail. We also utilized the CHESCA maps to assess the allosteric communication among the communities as defined by McClendon *et al*.^37^ Using the community definition, the CHESCA map for PKA-C^WT^ shows a strong correlation across the enzyme, especially for structurally adjacent communities and at the interface between the two lobes (see for instance the correlations among ComA, ComB, ComC, ComE, and ComH). The community CHESCA for F100A shows that the cross-talk between the nucleotide-binding (ComA) and positioning of αC-helix (ComB) is preserved, as well as the coupling between the R-spine assembly (ComC), and the activation loop (ComF). Except for ComD, the correlations among these communities in the N-lobe and ComF1, ComG, and Com H are completely abolished. Note that ComF1, ComG, and ComH are involved in the substrate binding and are instrumental in docking the R-subunits. Overall, the CHESCA analysis shows that the reduced degree of cooperativity we determined thermodynamically for PKA-C^F100A^ corresponds to a decrease in coordinated structural changes upon ligand binding, and these changes affect the C-lobe communities involved in the substrate binding and protein/protein interactions.

## DISCUSSION

A distinct feature of PKA-C is the binding cooperativity between nucleotide and substrate that originate from the allosteric coupling between the nucleotide-binding pocket and the interfacial region between the two lobes that harbors the substrate binding cleft. Structural and dynamic NMR data suggested that a well-tuned coupling between the two lobes of the kinase is required for efficient substrate binding. Also, additional NMR and functional studies showed that mutations in the activation loop linked to Cushing's syndrome reduce drastically substrate binding affinity, and more importantly, reduce the communication between the ligand binding pockets.^14–16^ Interestingly, a mutation (E31V) distal from the active site and linked to the progression of Cushing's syndrome has a similar effect, suggesting a possible allosteric modulation of the kinase substrate recognition.^16^ NMR chemical shift perturbation data suggested that these mutations are connected to allosteric nodes that, once perturbed, radiate their effects in the periphery of the enzyme and prevent an efficient dynamic coupling between the two lobes of the enzyme. Interestingly, these mutations do not prevent Kemptide phosphorylation. Rather, they cause a loss of substrate fidelity with consequent aberrant phosphorylation of downstream substrates.^38–43^ Additionally, thermodynamics and recent NMR studies using different nucleotides and inhibitors demonstrated that it is possible to control substrate binding affinity by changing the chemistry of the ligand at the ATP binding pocket. Altogether, these studies suggest that phosphorylation reaction and binding synergy between ATP and substrates may be controlled independently.

Our NMR data combined with RAM simulations and MSM enabled us to comprehensively map the free energy landscape of PKA-C in various forms. We found that the active kinase unleashed from the regulatory subunits occupies a broad energy basin (GS) that corresponds to the conformation of the ternary structure of PKA-C with ATP and pseudosubstrate (PKI_5–24_) that exemplifies a catalytic competent state, poised for phosphoryl transfer.^44^ We also found two orthogonal conformationally excited states ES1 and ES2. While the ES1 state corresponds to the inactive kinase conformations, the ES2 state was never observed in the crystallized structures. Our previous CEST NMR measurements suggested the presence of a sparsely populated state that, at that time, we were unable to characterize structurally. These new simulations and MSM show that the transition from GS to ES2 is due to a disruption of hydrophobic packing, featuring a conformational rearrangement for the αC-β4 loop, which causes a partial disruption of the hydrophobic R-spine. These structural changes interrupt the allosteric coupling between the two lobes, as shown by mutual information analysis. A single mutation (F100A), suggested by our simulations, promotes the flip of the αC-β4 loop and reproduces the hypothesized structural uncoupling between the two lobes of PKA-C. We experimentally tested the effects of the F100A mutations and found that it prevents the enzyme from adopting a conformation competent for substrate binding, resulting in a drastic reduction of the cooperativity between ATP and nucleotide. These NMR data further support our working model, showing that the inter-lobe communication is interrupted and the binding response of the F100A kinase is attenuated based on chemical shift perturbation. Furthermore, our study further emphasizes the active role of the hydrophobic interior of the kinase and shows that small alterations in the spines and shell sequences may lead to a dysfunctional kinase by either preventing phosphorylation (see V104G and I150A mutations)^27^ or by disrupting the binding cooperativity as for F100A.

The αC-β4 loop is a regulatory element present in all EPKs and its importance has been stressed in bioinformatics studies^45^ and supported by computational work, showing that F100 and F102 are at intersections of various communities and constitute a critical hydrophobic node, anchoring the N- to the C-lobe.^37^ More importantly, studies on EGFR and ErbB2 kinases lead to the hypothesis that the αC-β4 loop may act as a molecular brake ^46,47^ or an autoinhibitory switch.^48^ Therefore, it is not surprising that activating mutations and in-frame insertions in the αC-β4 loop are frequently found in kinase-related cancers, such as somatic oncogenic mutations such as P101S, P101L, and L103F.^49^ Finally, an elegant study by Kannan and coworkers emphasized the role of the αC-β4 loop in dimerization of EGFR.^21^ These researchers found that in-frame insertions rigidify and activate the kinase in a length dependent manner. More importantly, these human mutations display a gradual response to drugs, a factor that may exploited for designing mutant-selective inhibitors of EGFR.^50^ The data presented here show that it is possible to abolish the binding cooperativity of a kinase by turning the dial in the opposite direction, *i.e.*, increasing the dynamics of the αC-β4 loop and disconnecting the allosteric network between the N- and C-lobes. The identification of this new, partially inactivating pathway provides further understanding of how to control the dynamics and function of kinases.

## MATERIAL AND METHODS

### Replica-averaged metadynamics (RAM) simulations

#### I. System setup.

We used the crystal structure of the wild type PKA-C (PDB ID: 1ATP) as the template and added the missing residues 1–14 at the N terminus. The protonation state of histidine residues followed our previous settings.^51^ The protein was solvated in a rhombic dodecahedron solvent box with the tree-point charge TIP3P model^52^ for water extended approximately 10 Å away from the surface of the proteins. Counter ions (K^+^ and Cl^−^) were added to ensure electrostatic neutrality corresponding to an ionic concentration of ~ 150 mM. All protein covalent bonds were constrained with the LINCS algorithm.^53^ and long-range electrostatic interactions are treated with the particle-mesh Ewald method with a real-space cut-off of 10 Å.^54^ Parallel simulations on the apo form, the binary form with one Mg^2+^ ion and one ATP, and the ternary form with two Mg^2+^ ions, one ATP and one PKI_5–24_ are performed simultaneously using GROMACS 4.6^55^ with CHARMM36a1 force field.^56^ For the two mutants, F100A and V104G, the corresponding residues were mutated through the mutagenesis wizard of PYMOL.

#### II. Standard MD simulations.

Each system was minimized using the steepest decent algorithm to remove the bad contacts, and then gradually heated to 300 K at a constant volume over 1 ns, using harmonic restraints with a force constant 1000 kJ/(mol *Å^2^) on heavy atoms of both proteins and nucleotides. Over the following 12 ns of simulations at constant pressure (1 atm) and temperature (300 K), the restraints were gradually released. The systems were equilibrated for an additional 20 ns without positional restraints. A Parrinello-Rahman barostat^57^ was used to keep the pressure constant, while a V-rescale thermostat ^58^ with a time step of 2 fs was used to keep the temperature constant. Each system was simulated for 1.05 µs, with snapshots recorded every 20 ps.

#### III. Replica exchange (REX) simulations.

Following the standard MD simulations, parallel replica exchange (REX) simulations on the apo, binary and ternary form of PKA-C were set up. For each REX simulations, four replicas were used, and the initial structures were randomly chosen from the µs-scale unbiased simulations. Chemical shifts of PKA-C for N, CA, CO, CB, HN from NMR experiments were imposed as restraints based on the penalty function

$$E^{CS}=\alpha \sum_{k=1}^{N}\sum_{l=1}^{5}{\left(\delta_{kl}^{exp}-\frac{1}{M}\sum_{m=1}^{M}\delta_{kl}^{calc}\right)}^{2}$$

where α is the force constant, k runs over all residues of the protein, l denotes the different backbone atoms, and m runs over the four replicas. $\delta_{kl}^{calc}$ is computed using CamShift that is a module of ALMOST-2.1.^59^ The force constant was gradually increased from 0 (unbiased) to 20 (maximum restraints for production) over 50 ns. All other settings are the same as the previous unbiased simulations. REX simulations were carried out with GROMACS 4.6,^55^ with the replica exchange controlled by the module PLUMED 2.1.1.^60^ These simulations were further conducted ~100 ns for each replica of the three forms.

#### IV. Replica-averaged metadynamics (RAM) simulations.

The subsequent RAM simulations were started from the final structures of REX simulations. The CS restraints were imposed in the same way as the REX simulations. Four CVs are chosen to increase the conformational plasticity around the catalytic cores (detailed in Figure S1): (CVI) the ψ angles of backbone of all the loops that are not in contact with ATP (Back-far), (CVII) the ϕ angles of backbone of all the loops that are in contact with ATP (Back-close), (CVIII) the χ_1_ angles of side-chains of all the loops that are in contact with ATP (Side-close), (CVIV) the radius of gyration calculated over the rigid part (*i.e.*, residues 50–300) of the protein (rgss). Gaussian deposition rate was performed with an initial rate of 0.125 kJ/mol/ps, where the σ values were set to 0.5, 0.2, 0.2, and 0.01 nm for the four CVs, respectively. The RAM simulations were also carried out with GROMACS 4.6 in conjunction with PLUMED 2.1 and ALMOST 2.1, and continued for ~400 ns for each replica, with exchange trails every 1 ps.

#### V. Reconstruction of free energy surface (FES) from the RAM simulations.

After about 300 ns, the sampling along the first 3 CVs reached convergence, allowing a reliable reconstruction of the corresponding FES. The production run was continued for another 100 ns to sample enough conformations. These conformations were first clustered into microstates using the regular spatial method (cut-off radius of 0.13), and then the free energy of each state is reweighted according to the deposited potential along each CV, which can be obtained from the analysis module of METAGUI.^61^ To visualize the distribution of these microstates and their relative energies differences, we further performed principal component analysis to project the microstates represented in the 3-dimennsional CV space into 2-dimensional space spanned by PC1 and PC2. Then we can plot these microstates in a 3-dimensional space spanned by PC1, PC2 and free energy differences *∆G*.

#### VI. Independent validation of chemical shifts with SPARTA+.

During the REX and RAM simulations, chemical shifts were computed via CamShift in ALMOST 2.1. As an independent validation for the efficacy of the bias, we further calibrated chemical shifts of 2000 snapshots with MDTraj^62^ and SPARTA+.^23^

#### VII. Adaptive sampling.

The first round of adaptive sampling started from the snapshots of low-energy microstates obtained from the previous step, *i.e.*, 1200 structures for the apo form, 400 structures for the binary form and 200 structures for the ternary form. The initial velocities were randomly generated to satisfy the Maxwell distribution at 300K. For the apo form, a 10 ns simulation was performed for each run, whereas for the binary, each simulation lasted 30 ns, resulting in a total of 12 μs trajectories for both the apo and binary forms. To obtain converged free energy landscape, a total of three rounds of adaptive sampling was started from the 400 microstates that was obtained by K-mean clustering of all snapshots of previous ensembles. Therefore, a total of 100 µs trajectories and 100,0000 snapshots (100 ps per frame) were collected for both the apo and binary form after three rounds of adaptive sampling, and a total of 60 µs trajectories were collected for the ternary form.

#### VIII. Markov state model (MSM) and time-lagged independent component analysis (tICA).

The Cartesian coordinates of key hydrophobic residues, include R-spine residues, L95, L106, Y164 and F185, and the shell residues, V104, M118 and M120, were chosen as the metrics to characterize the conformational transition of the hydrophobic core of PKA-C. Specifically, each snapshot was first aligned to the same reference structure by superimposition of αE (residues 140–160) and αF helices (residues 217–233), and represented by the deviation of Cartesian coordinates of the key residues. The representation in this metric space was further reduced to 10-dimension vectors using time-lagged independent component analysis (tICA)^63^ at a lag time of 1 ns. All the snapshots were clustered into 400 microstates with K-mean clustering. A MSM was built upon the transition counts between these microstates.

#### IX Kinetic Monte Carlo trajectory of PKA-C in different forms.

Long trajectories were generated using a kinetic Monte Carlo method based on the MSM transition probability matrix of the three forms of PKA-C. Specifically, the discrete jumps between the 100 microstates were sampled for 60 us. And then random conformations were chosen for each state from all the available snapshots. Time series of various order parameters were analyzed subsequently.

### Protein expression and purification

The recombinant human Cα subunit of cAMP-dependent protein kinase with the Phe to Ala mutation in position 100 (PKA-C^F100A^) was generated from the human PKA-Cα wild-type using QuikChange Lightning mutagenesis kit (Agilent genomics). The key resource table lists the PCR primers used to modify the pET-28a expression vector encoding the wild-type human PKA-Cα gene (*PRKACA –* uniprot P17612) ^14–16^. The unlabeled and uniformly ^15^N-labeled PKA-C^F100A^ mutant was expressed and purified following the same protocols used for the wild-type protein.^64^ Briefly, transformed *E. coli* BL21 (DE3) *pLyss* cells (Agilent) were cultured overnight at 30 °C in Luria-Bertani (LB) medium. The next morning, the cells were transferred to fresh LB medium for the overexpression of the unlabeled protein or to M9 minimal medium supplied with ^15^NH_4_Cl (Cambridge Isotope Laboratories Inc.) as the only nitrogen source for the labeled protein overexpression. In both cases, protein overexpression was induced with 0.4 mM of β-D-thiogalactopyranoside (IPTG) and carried out for 16 hours at 20 °C. The cells were harvested by centrifugation and resuspended in 50 mM Tris-HCl, 30 mM KH_2_PO_4_, 100 mM NaCl, 5 mM 2-mercaptoethanol, 0.15 mg/mL lysozyme, 200 μM ATP, DNaseI, 1 tablet of cOmplete ULTRA EDTA-free protease inhibitors (Roche Applied Science) (pH 8.0) and lysed using French press at 1,000 psi. The cell lysate was cleared by centrifugation (60,000 × g, 4 °C, 45 min), and the supernatant was batch-bound with Ni^2+^-NTA agarose affinity resin (Thermo Scientific). The his-tagged PKA-C^F100A^ was eluted with 50 mM Tris-HCl, 30 mM KH_2_PO_4_, 100 mM NaCl, 5 mM 2-mercaptoethanol, 0.5 mM phenylmethylsulfonyl fluoride (PMSF) (pH 8.0) supplied with 200 mM of imidazole. The tail of poly-His was cleaved using a stoichiometric amount of recombinant tobacco etch virus (TEV) protease in 20 mM KH_2_PO_4_, 25 mM KCl, 5 mM 2-mercaptoethanol, 0.1 mM PMSF (pH 6.5), overnight at 4 °C. The different phosphorylation states of PKA-C^F100A^ were separated using a cation exchange column (HiTrap Q-SP, GE Healthcare Life Sciences) using a linear gradient of KCl in 20 mM KH_2_PO_4_ at pH6.5.^65^ The purified protein isoforms were then stored in phosphate buffer containing 10 mM dithiothreitol (DTT), 10 mM MgCl_2_, and 1 mM NaN_3_ at 4 °C. The protein purity was assessed by sodium dodecyl sulfate-polyacrylamide gel electrophoresis (SDS–PAGE).

### Peptide synthesis

The Kemptide (LRRASLG) and PKI_5–24_ (TTYADFIASGRTGRRNAIHD) peptides were synthesized using a CEM Liberty Blue microwave synthesizer using standard Fmoc chemistry. All peptides were cleaved with Reagent K (82.5% TFA, 5% phenol, 5% thioanisole, 2.5% ethanedithiol, and 5% water) for 3 h and purified using a semipreparative Supelco C18 reverse-phase HPLC column at 3 mL/min.

The purified peptides were concentrated, lyophilized, and stored at −20 °C. Molecular weight and quantity were verified by MALDI-TOF and/or amino-acid analysis (Texas A&M University).

### Isotherm titration calorimetry (ITC) measurements

PKA-C^F100A^ was dialyzed into 20 mM MOPS, 90 mM KCl, 10 mM DTT, 10 mM MgCl_2_, and 1 mM NaN_3_ (pH 6.5) and concentrated using conical spin concentrator (10 KDa membrane cut-off, Millipore) to a solution at 80–100 μM, as confirmed by A280 = 55,475 M^−1^ cm^−1^. Approximately 300 μL of protein was used for each experiment, with 50 μL of 2 mM ATPγN and/or 1 mM PKI_5–24_ in the titrant syringe. All measurements were performed at 300 K in triplicates with a low-volume NanoITC (TA Instruments). The binding was assumed to be 1:1, and curves were analyzed with the NanoAnalyze software (TA Instruments) using the Wiseman isotherm ^32^

$$\frac{d\left[MX\right]}{d\left[X_{tot}\right]}=\Delta H^{\circ }V_{0}\left[\frac{1}{2}+\frac{1-\frac{1-r}{2}-R_{m}/2}{{\left(R_{m}^{2}-2R_{m}\left(1-r\right)+{\left(1+r\right)}^{2}\right)}^{1/2}}\right]$$

where $d\left[MX\right]$ is the change in total complex relative to the change in total protein concentration, $d\left[X_{tot}\right]$ is dependent on r (the ratio of $K_{d}$ relative to the total protein concentration), and $R_{m}$ (the ratio between total ligand and total protein concentration). The heat of dilution of the ligand into the buffer was considered for all experiments and subtracted.

The free energy of binding was determined from:

$$\Delta G=RT\ln K_{d}$$

where R is the universal gas constant and T is the temperature at measurement (300 K). The entropic contribution to binding was calculated using:

TΔS=ΔH−ΔG

The degree of cooperativity $\left(\text{σ}\right)$ was calculated as:

$$\sigma =\frac{K_{d\;apo}}{K_{d\;nucleotide}}$$

where $K_{d\;apo}$ is the dissociation constant of PKI_5–24_ binding to the apo-enzyme, and $K_{d\;Nucleotide}$ is the corresponding dissociation constant for PKI_5–24_ binding to the nucleotide-bound the kinase.

### Enzyme assays

Steady-state coupled enzyme activity assays using Kemptide as substrate were performed under saturating ATP concentrations and spectrophotometrically at 298 K, as reported by Cook *et al.*^30^ The values of $V_{max}$ and $K_{M}$ were obtained from a nonlinear fit of the initial velocities to the Michaelis-Menten equation.

### NMR spectroscopy

NMR measurements were performed on a Bruker Avance NEO spectrometer operating at a ^1^H Larmor frequency of 600 MHz equipped with a cryogenic probe or on a Bruker Avance III 850 MHz spectrometer equipped with a TCI cryoprobe. The NMR experiments were recorded at 300K in 20 mM KH_2_PO_4_ (pH 6.5), 90 mM KCl, 10 mM MgCl_2_, 10 mM DTT, 1 mM NaN_3_, 5% D_2_O, and 0.1% 4-benzene sulfonyl fluoride hydrochloride (AEBSF, Pefabl–c - Sigma-Aldrich). Concentrations for samples were 0.15 mM of uniformly ^15^N-labeled PKA-C^F100A^, as determined by A_280_ measurements, 12 mM ATPγN or ADP was added for the nucleotide-bound form, and 0.3 mM PKI_5–24_ for the ternary complex. [^1^H, ^15^N]-WADE-TROSY-HSQC pulse sequence ^66^ was used to record the amide fingerprint spectra of PKA-C^F100A^ in the apo, nucleotide-bound (ADP- or ATPγN-bound – binary form), and ternary complex (PKAC^F100A^/ATPγN/PKI_5–24_). All [^1^H, ^15^N]-WADE-TROSY-HSQC experiments were acquired with 2048 (proton) and128 (nitrogen) complex points, processed using NMRPipe^67^ and visualized using NMRFAM-SPARKY ^68^ and POKY.^69^ Combined chemical shift perturbations (CSP) were calculated using ^1^H and ^15^N chemical shifts according to:

$$\Delta \delta =\sqrt{{\left(\Delta \delta H\right)}^{2}+{\left(0.154\times \Delta \delta N\right)}^{2}}$$

in which Δδ is the CSP; $\Delta {\text{δ}}_{H}$ and $\Delta {\text{δ}}_{N}$ are the differences of ^1^H and ^15^N chemical shifts, respectively, between the first and last point of the titration; and 0.154 is the scaling factor for nitrogen.^70^

#### COordiNated ChemIcal Shift bEhavior (CONCISE).

The normal distributions reported in the CONCISE plot were calculated using principal component analysis for residues whose chemical shifts responded linearly to ligand binding.^34^ In this work, we use the ^1^H and ^15^N chemical shifts derived from the [^1^H, ^15^N]-WADE-TROSY-HSQC experiments for the apo, ADP, ATPγN, and ATPγN/PKI_5–24_ bound forms of PKA-C.

#### CHEmical Shift Covariance Analysis (CHESCA).

This analysis was used to identify and functionally characterize allosteric networks of residues eliciting concerted responses to, in this case, nucleotide and pseudosubstrate. To identify inter-residue correlations, four states were used: apo, ATPγN-bound, ADP-bound, and ATPγN/PKI_5–24_. The identification of inter-residue correlations by CHESCA relies on agglomerative clustering (AC) and singular value decomposition (SVD). Pairwise correlations between chemical shift variations experienced by different residues were calculated to identify networks. When plotted on a correlation matrix, this allows for the identification of regions that are correlated to one another. For each residue, the maximum change in chemical shift was calculated in both the ^1^H (*x*) and ^15^N (*y*) dimensions $\left(\Delta {\text{δ}}_{x,y}\right)$. The residues included in the CHESCA analysis were the ones that satisfied the following: Δδx,y>12ΔνxA,yA+12ΔνxB,yB, where A and B correspond to two different forms analyzed (note that there is no dependence on which two forms satisfied this statement), and ∆ν denotes the linewidth. Correlation scores were used to quantify the CHESCA correlation of a single residue or a group of residues with another group. Correlation scores were evaluated for both (a) a single residue and (b) the full protein. The generalized expression for evaluating either case is:

$$Corr\;Score=\frac{number\;of\;\left(R_{ij}>R_{cutoff}\right)}{total\;number\;of\;R_{ij}}$$

where $R_{iJ}$ is the CHESCA correlation matrix and i and j denote (a) a single residue and all other assigned residues in the protein, respectively, or (b) both represent all the assigned residues in the protein. For all the analysis a $R_{cutoff}$ of 0.98 was used.

Community CHESCA analysis is a chemical shift-based correlation map between functional communities within the kinase. Each community is a group of residues ^37^ associated with a function or regulatory mechanism. To represent community-based CHESCA analysis, we lowered the correlation cut-off such that $R_{cutoff}>0.8$.

Suppose communities X and Y have $n_{A}$ and $n_{B}$ number of assigned residues respectively, the correlation score between A and B is defined as,

$$R_{A,B}=number\;of\left(R_{ij}>R_{cutoff}\right)/\left(n_{A}*n_{B}\right)$$

where $R_{iJ}$ is the CHESCA correlation coefficient between residue i (belongs to community A) and residue j (belongs to community B). $R_{cutoff}$ is the correlation value cutoff. $R_{A,B}$ can take values from 0 (no correlation between residues in A and B) to 1 (all residues in A has correlation > cutoff with all residues in B).

## Supplementary Material

Supplement 1

## Figures and Tables

**Table:** Key Resources Table

Reagent type (species) or resource	Designation	Source or reference	Identifiers	Additional information
Gene (*Homo sapiens)*	PKA-Cα		PKA-C	Uniprot ID: P17612
strain, strain background (*Escherichia coli*)	BL21(DE3) pLyss	Agilent	Cat. #200132	Chemically competent cells
Sequence-based reagent	PKA-C^F100A^	This study	PCR primer (Forward)	tattctgcaagcggtg aacgccccgtttctg gttaagctg
Sequence-based reagent	PKIa 5–24	Synthetic peptide	PKI_5–24_	Chemically synthesized
Sequence-based reagent	Kemptide ()	Synthetic peptide	Kemptited	LRRASLG
Commercial assay or kit	QuikChange Lightning Multi Mutagenesis Kit	Agilent genomics	Cat #210519	Commercial mutagenesis kit
Recombinant DNA reagent	PKA-^CF100A^	This study	PKA-C^F100A^	Single Ala mutant of PKA-C
Chemical compound, drug	AMP-PNP or ATPγN	Roche Applied Science	CAS 25612-73-1	ATP analogous
Chemical compound, drug	ADP	Sigma-Aldrich	CAS 20398-34-9	nucleotide
Software, algorithm	TopSpin 4.1	Bruker Inc.	https://www.bruker.com/	
Software, algorithm	NMRFAM-Sparky	NMRFam	https://nmr-fam.wisc.edu/nmr-fam-sparky-distribution/	
Software, algorithm	NMRPipe	Delaglio F, NIH	https://www.ibbr.umd.edu/nmrpipe/install.html	
Software, algorithm	POKY	Lee W,	https://sites.google.com/view/pokynmr	
Software, algorithm	COordiNated ChemIcal Shift bEhavior (CONCISE)	Veglia G	https://conservancy.umn.edu/handle/11299/217206 https://conservancy.umn.edu/handle/11299/227294	Matlab script
Software, algorithm	CHEmical Shift Covariance Analysis (CHESCA)	Melacini G	https://academic.oup.com/bioinformatics/article/37/8/1176/5905475?login=true	NMRFAM-Sparky&POKY tools
Software, algorithm	PyMol	Schrödinger, LLC	https://pymol.org	
Software, algorithm	MatLab2022b	MathWorks	https://www.mathworks.com/products/matlab.html	
Software, algorithm	GraphPad Prism 9	GraphPad Software Inc.	https://www.graphpad.com/	
Software, algorithm	GROMACS 4.6	Hess B et al	http://www.gromacs.org/	
Software, algorithm	CHARMM36a 1	Best RB et al (2012)	https://pubs.acs.org/doi/10.1021/ct300400x	
Software, algorithm	PLUMED 2.1.1	Bonomi M, et al. (2009)	https://www.plumed.org/doc-v2.5/user-doc/html/_c_h_a_n_g_e_s-2-1.html	
Software, algorithm	ALMOST 2.1	Kohlhoff et al (2009).	svn://svn.code.sf.net/p/almost/code/almost-code	
Software, algorithm	METAGUI	Biarnés X et al. (2012)	https://www.sciencedirect.com/science/article/pii/S0010465511003079	
Software, algorithm	MDTraj	McGibbon RT et al (2015)	https://www.sciencedirect.com/science/article/pii/S0006349515008267	
Software, algorithm	SPARTA+	Yang Shen, Ad Bax (2010)	https://spin.niddk.bov/bax/software/SPARTA+/	
Software, algorithm	MSMbuilder	Beauchamp KA et al (2011)	https://msmbuilder.org/	
Software, algorithm	Mutinf	Mcclendon C et al (2012)	https://simtk.org/projects/mutinf/	
