# Supplementary material for: The αC-β4 loop controls the allosteric cooperativity between nucleotide and substrate in the catalytic subunit of protein kinase A
Source: bioRxiv. 2023 Sep 15:2023.09.12.557419. Preprint. [Version 1] doi: 10.1101/2023.09.12.557419 (PMC10515842; doi:10.1101/2023.09.12.557419)
Supplement: Supplement 1 [file NIHPP2023.09.12.557419v1-supplement-1.pdf]

bioRxiv preprint doi: https://doi.org/10.1101/2023.09.12.557419; this version posted September 15, 2023. The copyright holder for this preprint (which was not certified by peer review) is the author/funder, who has granted bioRxiv a license to display the preprint in perpetuity. It is made available under aCC-BY 4.0 International license.

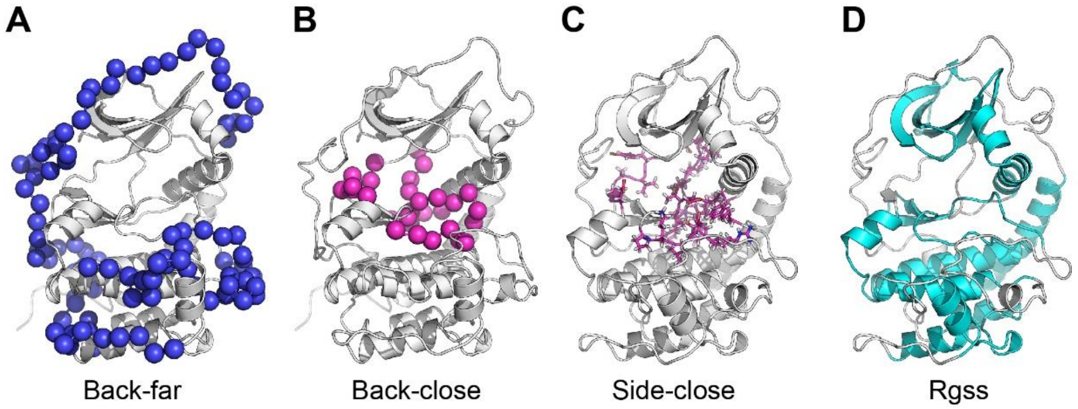

Figure 2 – figure supplement 1. **Illustration of the collective variables (CVs) used in the RAM simulations.** (**A**) The ψ angles of the backbone of all the loops not in contact with ATP (Back-far), where the Cα atoms of the residues involved are highlighted in the blue sphere. (**B**) The ψ angles of the backbone of all the loops in contact with ATP (Back-close), where the Cα atoms of the residues involved are highlighted in magenta sphere. (**C**) The χ1 angles of side chains of all the loops that are in contact with ATP (Side-close), where the side chains of the residues involved are highlighted in magenta stick. (**D**) The radius of gyration is calculated over the rigid part of the protein (rgss), where the residues involved are colored in cyan.

bioRxiv preprint doi: https://doi.org/10.1101/2023.09.12.557419; this version posted September 15, 2023. The copyright holder for this preprint (which was not certified by peer review) is the author/funder, who has granted bioRxiv a license to display the preprint in perpetuity. It is made available under aCC-BY 4.0 International license.

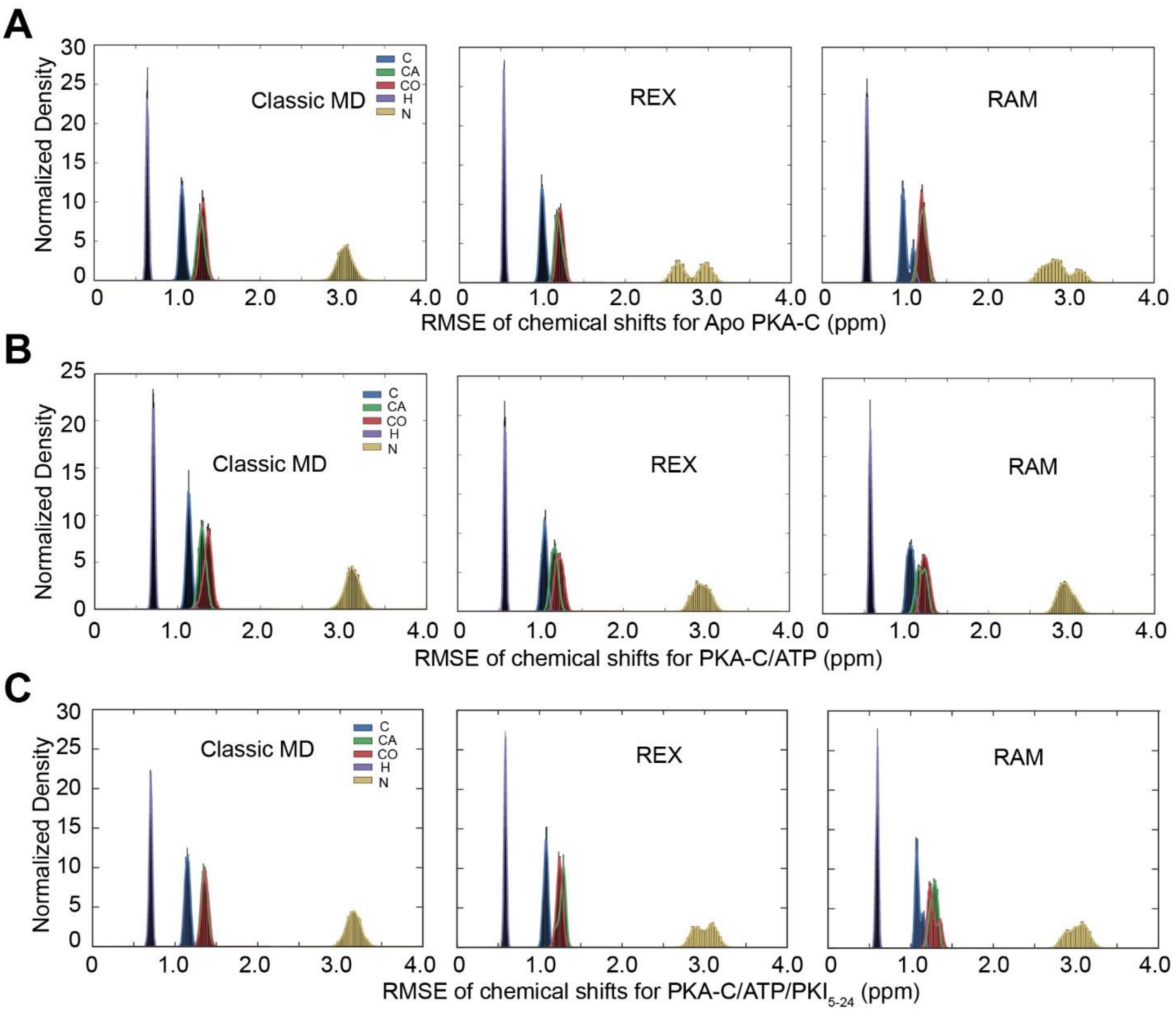

Figure 2 – figure supplement 2. **Distribution of the Root-Mean-Square-Error (RMSE) of the chemical shifts in different simulation schemes**. (**A**) RMSE of CS for the apo PKA-C from standard MD (left), REX (middle), and RAM (right). (**B**) RMSE of CS for PKA-C/ATP from standard MD (left), REX (middle), and RAM (right). (**C**) RMSE of CS for PKA-C/ATP/PKI$_{5\text{-}24}$ from standard MD (left), REX (middle), and RAM (right). Color codes for different backbone atoms (C, Cα, CO, H and N) are shown in the left figures.

bioRxiv preprint doi: https://doi.org/10.1101/2023.09.12.557419; this version posted September 15, 2023. The copyright holder for this preprint (which was not certified by peer review) is the author/funder, who has granted bioRxiv a license to display the preprint in perpetuity. It is made available under aCC-BY 4.0 International license.

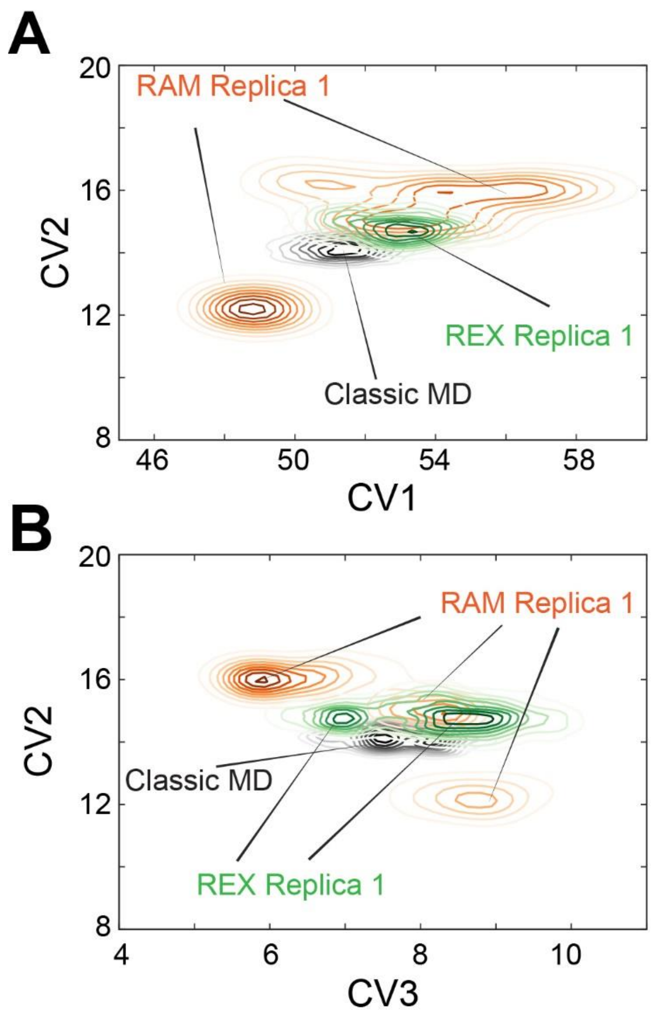

Figure 2 – figure supplement 3. **Replica-averaged metadynamics (RAM) simulations explore a larger conformational space than standard MD and replica exchange (REX) simulations**. (**A**) Comparison of conformational space sampled by RAM Replica 1, standard MD, and REX Replica 1 of the apo PKA-C, along the CV1 and CV2. (**B**) Comparison of conformational space sampled by RAM Replica 1, standard MD, and REX Replica 1 of the apo PKA-C, along the CV3 and CV2.

bioRxiv preprint doi: https://doi.org/10.1101/2023.09.12.557419; this version posted September 15, 2023. The copyright holder for this preprint (which was not certified by peer review) is the author/funder, who has granted bioRxiv a license to display the preprint in perpetuity. It is made available under aCC-BY 4.0 International license.

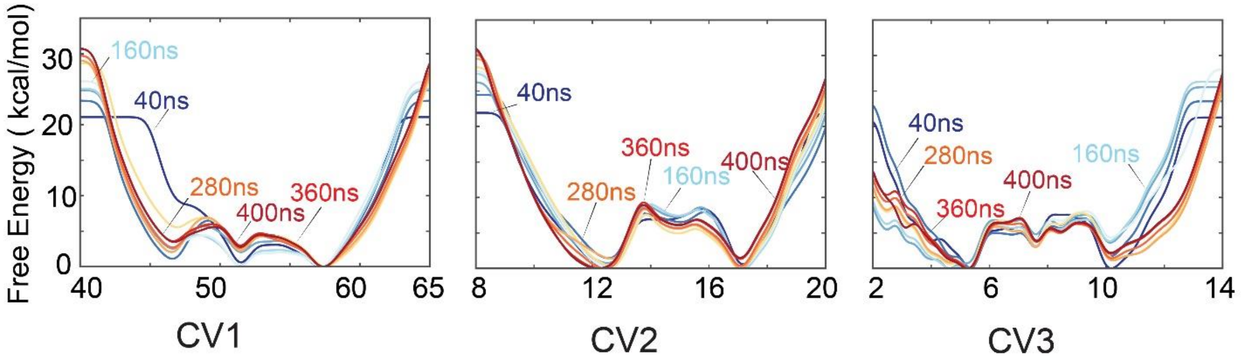

Figure 2 – figure supplement 4. Accumulative deposition of history-dependent biases along the first three CVs for the RAM simulation of the apo PKA-C. The accumulative biased converged after around 300 ns along all three CVs.

bioRxiv preprint doi: https://doi.org/10.1101/2023.09.12.557419; this version posted September 15, 2023. The copyright holder for this preprint (which was not certified by peer review) is the author/funder, who has granted bioRxiv a license to display the preprint in perpetuity. It is made available under aCC-BY 4.0 International license.

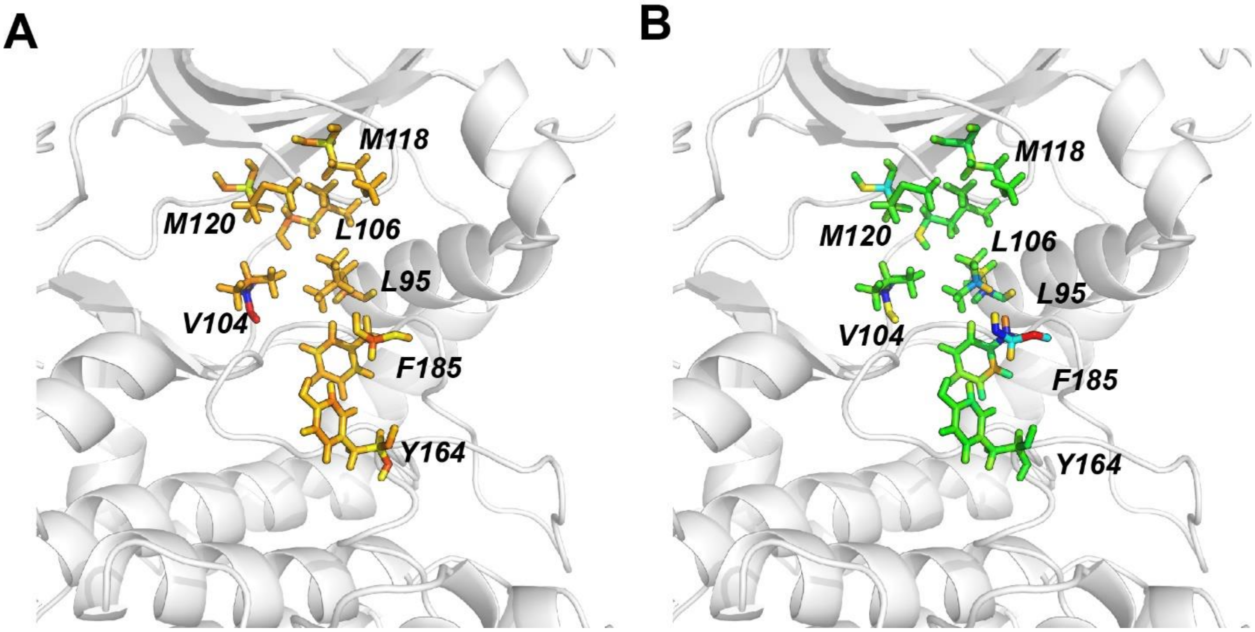

Figure 3 – figure supplement 1. **Residues of the regulatory spine and shell are chosen as the metrics for two time-lagged independent components (tICA) and Markov State Model (MSM) analysis.** (**A**) Atom motions of key residues that define tIC1 of the apo PKA-C, colored by the superposition deviations. Backbone atoms of Val104 show the largest change in tIC1. (**B**) Atom motions of key residues that define tIC2 of the apo PKA-C, colored by the superposition deviations. Backbone atoms of Phe185 and Val104 show largest change in tIC2.

bioRxiv preprint doi: https://doi.org/10.1101/2023.09.12.557419; this version posted September 15, 2023. The copyright holder for this preprint (which was not certified by peer review) is the author/funder, who has granted bioRxiv a license to display the preprint in perpetuity. It is made available under aCC-BY 4.0 International license.

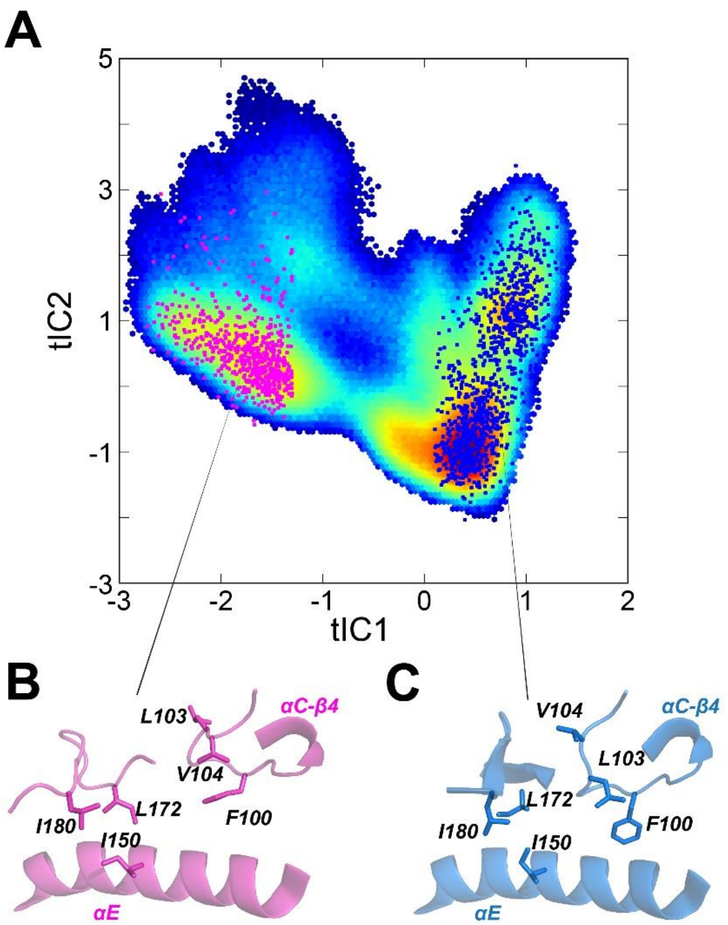

Figure 3 – figure supplement 2. **ES and GS in the apo PKA-C show distinct hydrophobic packing for residues around the αC-β4 loop.** (**A**) Projections of randomly selected conformations for ES (magenta) and GS (blue) onto the conformational landscape of the apo PKA-C. To best separate ES from GS, snapshots with tIC1 < 1.2 were clustered as ES, whereas those with tIC1 > 0.2 were clustered as GS. **(B,C)** Representative structure of ES (**B**) reveals different hydrophobic packing from that of GS (**C**), highlighted by the distinction at Leu103, Val104, Ile150, Leu172, and Ile180, where all show slow chemical exchanges in CPMG experiment of the apo PKA-C.

bioRxiv preprint doi: https://doi.org/10.1101/2023.09.12.557419; this version posted September 15, 2023. The copyright holder for this preprint (which was not certified by peer review) is the author/funder, who has granted bioRxiv a license to display the preprint in perpetuity. It is made available under aCC-BY 4.0 International license.

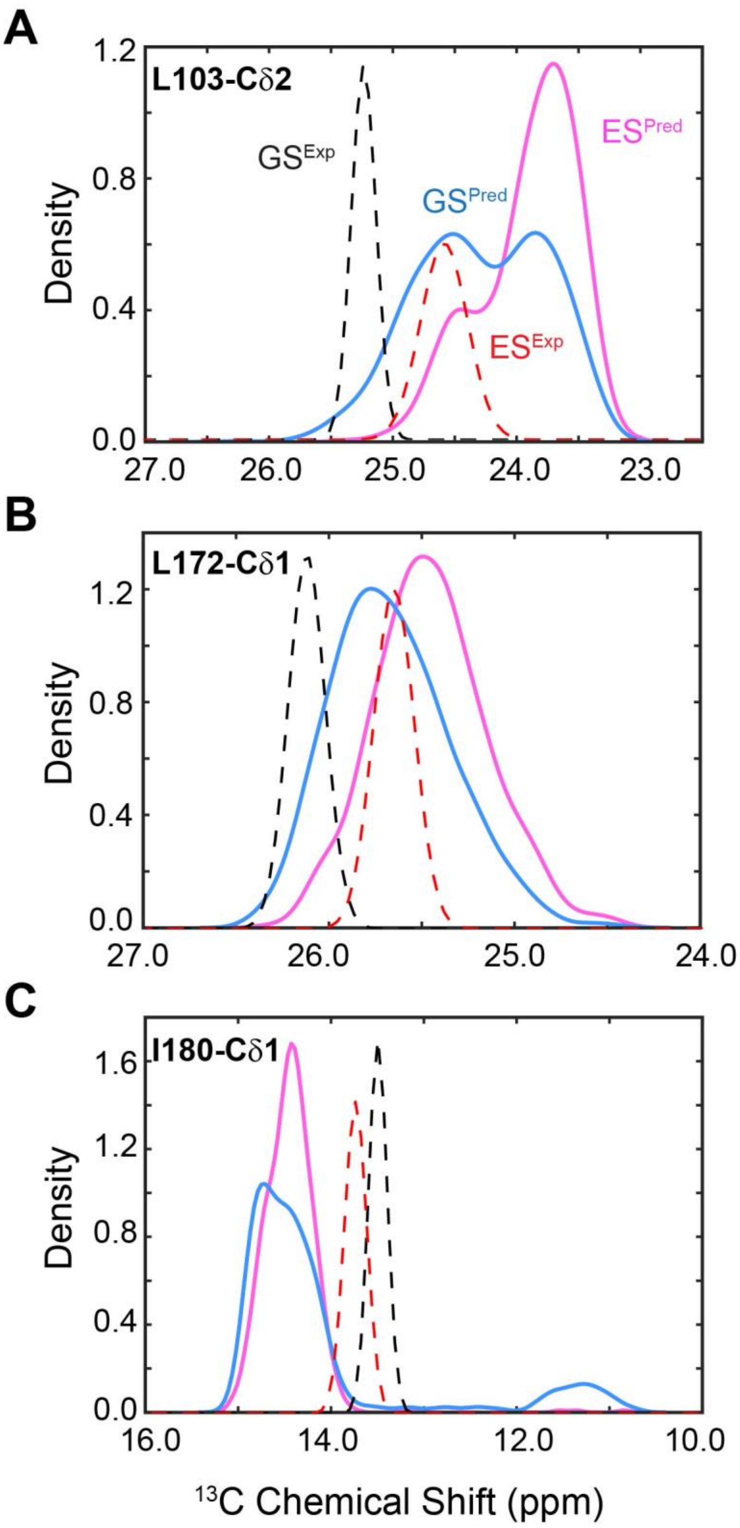

Figure S7. **Distribution of predicted ^{13}C CS of selected methyl groups**. **(A-C)** ES (magenta) and GS (blue) of the apo PKA-C for Leu103-Cδ2 (**A**), Leu172-Cδ1 (**B**) and Ile180-Cδ1 (**C**). The experimental CS are shown in dotted line for GS (black) and ES (red).

bioRxiv preprint doi: https://doi.org/10.1101/2023.09.12.557419; this version posted September 15, 2023. The copyright holder for this preprint (which was not certified by peer review) is the author/funder, who has granted bioRxiv a license to display the preprint in perpetuity. It is made available under aCC-BY 4.0 International license.

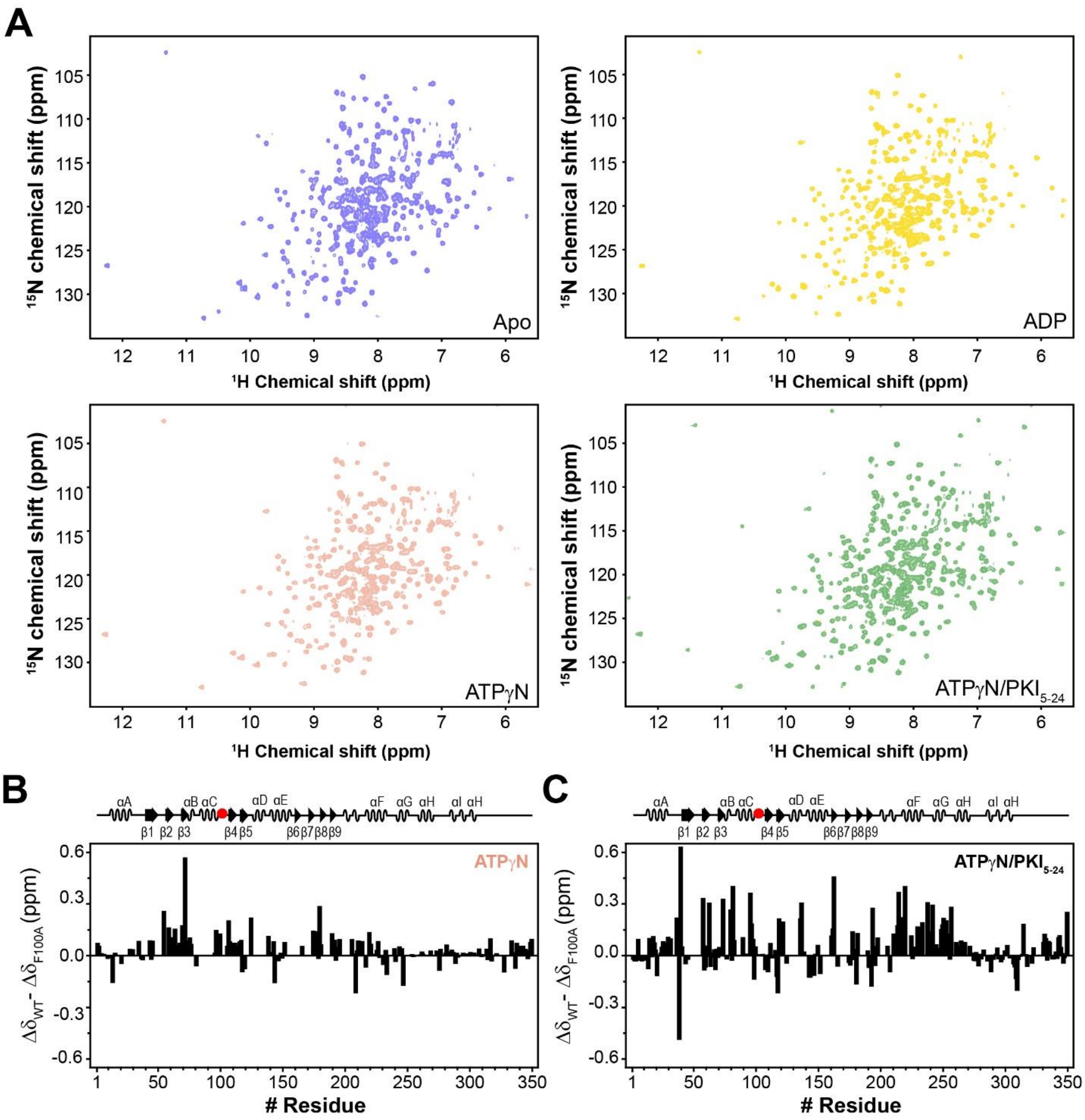

Figure S8 - figure supplement 1. NMR fingerprints of PKA-C^{F100A}. (**A**) [^{1}H,^{15}N]-WADE-TROSY spectrum of apo PKA-C^{F100A} and bound to bound to ADP, ATPγN, and e ATPγN/PKI$_{5-24}$. (**B**) Change in chemical shift perturbation (CSP) between PKA-C^{WT} and PKA-C^{F100A} upon binding ATPγN. (**C**) Change in CSP ($\Delta\delta_{WT}$ - $\Delta\delta_{F100A}$) upon binding ATPγN and PKI$_{5-24}$.

bioRxiv preprint doi: https://doi.org/10.1101/2023.09.12.557419; this version posted September 15, 2023. The copyright holder for this preprint (which was not certified by peer review) is the author/funder, who has granted bioRxiv a license to display the preprint in perpetuity. It is made available under aCC-BY 4.0 International license.

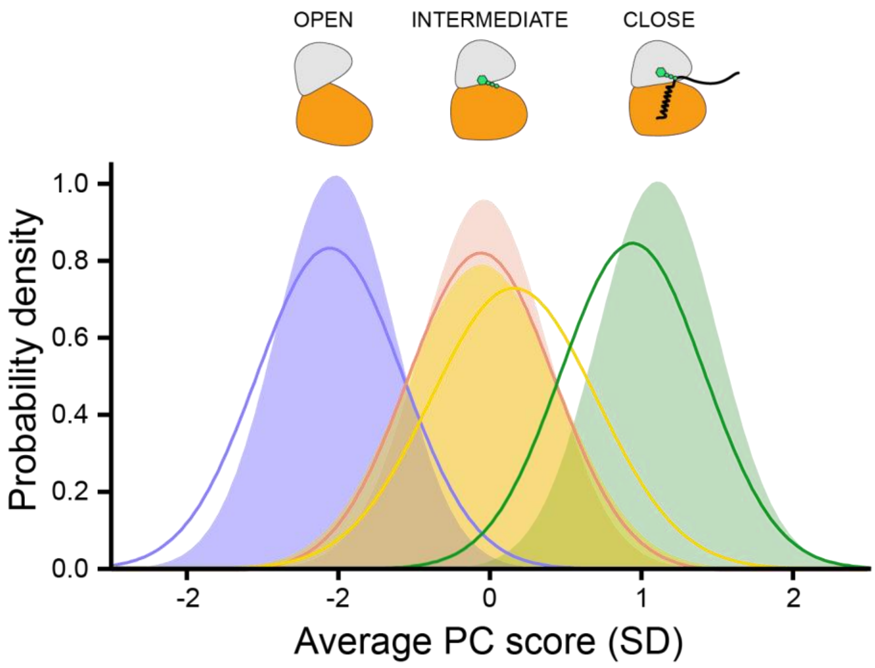

Figure S10 - figure supplement 1: CONCISE plot showing the probability distribution of the amide resonances as a function of ligand binding. The per-residue information is averaged into the average principal component (PC) score indicative of the position of each conformational state of the kinase along the equilibrium.

bioRxiv preprint doi: https://doi.org/10.1101/2023.09.12.557419; this version posted September 15, 2023. The copyright holder for this preprint (which was not certified by peer review) is the author/funder, who has granted bioRxiv a license to display the preprint in perpetuity. It is made available under aCC-BY 4.0 International license.

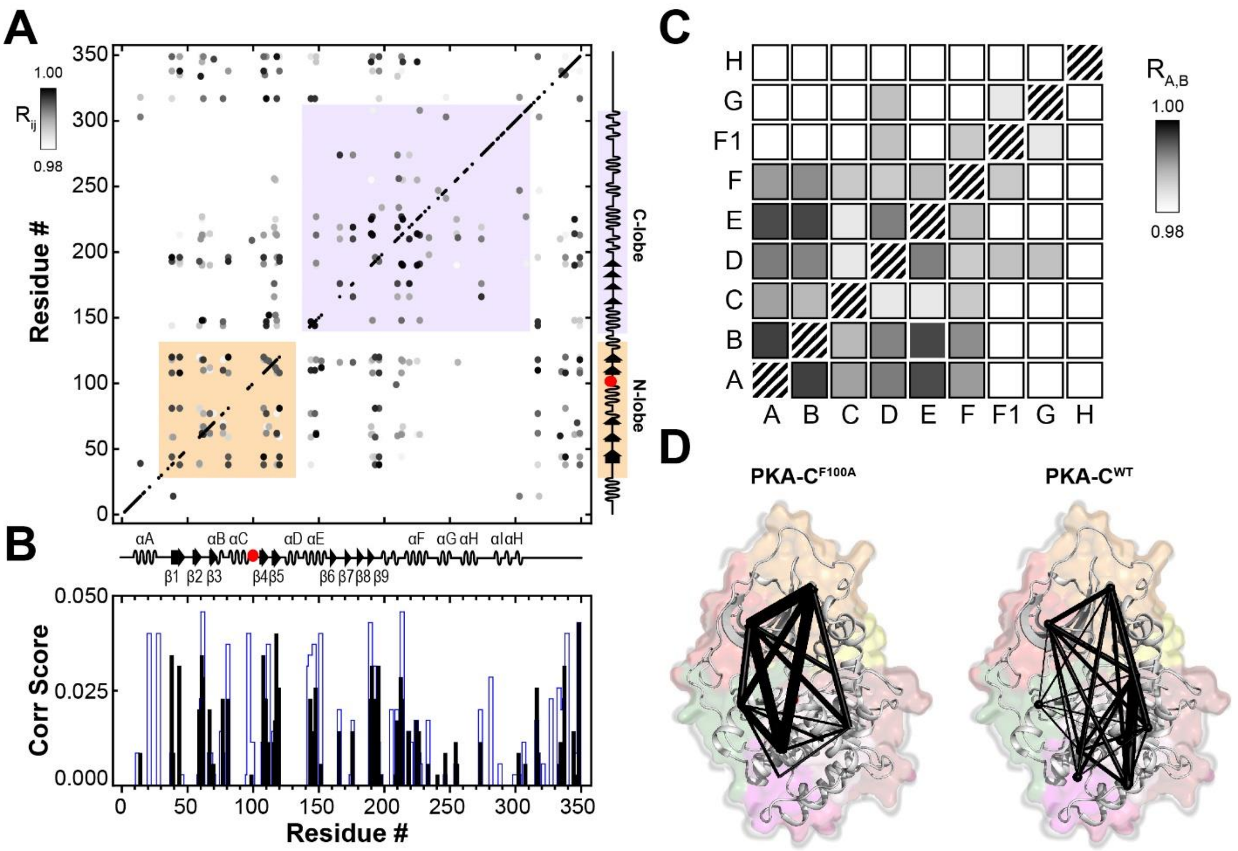

Figure S10 - figure supplement 2. Changes of the intermolecular allosteric network in F100A as mapped by correlated chemical shift changes. (A) CHESCA matrix obtained from the amide chemical shifts of PKA-CF100A in the apo, ADP-bound, ATPγN-bound, and ATPγN/$PKI_{5\text{-}24}$-bound states. Only correlations with $R_{ij} > 0.98$ are reported. **(B)** Plot of the correlation score vs. residue calculated for PKA-C^{WT} (blue) and PKA-C^{F100A} (black). **(C)** Community CHESCA analysis of and PKA-C^{F100A} (bottom diagonal, black). Only correlations with $R_{A,B} > 0.98$ are shown. (**D**) Community CHESCA matrices of PKA-C^{F100A} and PKA-C^{WT} plotted on their corresponding structures. The size of each node is independent of the number of residues it encompasses, meanwhile the weight of each line indicates the strength of coupling between the individual communities.

bioRxiv preprint doi: https://doi.org/10.1101/2023.09.12.557419; this version posted September 15, 2023. The copyright holder for this preprint (which was not certified by peer review) is the author/funder, who has granted bioRxiv a license to display the preprint in perpetuity. It is made available under aCC-BY 4.0 International license.

Figure 2 – supplementary table 1. ∆G (kcal/mol) and relative population of ground state and the first 6 excited states in different forms of PKA-C by the RAM simulations.

	GS	ES1	ES2	ES3	ES4	ES5	ES6
Apo	0 (58.0%)	0.38 (30.8%)	1.22 (7.6%)	2.10 (1.8.%)	2.28 (1.3%)	2.85 (0.5%)	8.16* (<1e-4)
Binary	0 (99.4%)	3.11 (0.6%)	5.80 (<1e-4)	6.66 (<1e-4)	7.25 (<1e-4)	8.27 (<1e-4)	8.47 (<1e-4)
Ternary	0 (100.0%)	4.85 (<1e-4)	5.68 (<1e-4)	6.92 (<1e-4)	7.04 (<1e-4)	7.58 (<1e-4)	7.87 (<1e-4)

* Numbers in red refer to populations of excited states below 0.5%.

bioRxiv preprint doi: https://doi.org/10.1101/2023.09.12.557419; this version posted September 15, 2023. The copyright holder for this preprint (which was not certified by peer review) is the author/funder, who has granted bioRxiv a license to display the preprint in perpetuity. It is made available under aCC-BY 4.0 International license.

Supplementary Table 2. Kinetic parameters of Kemptide phosphorylation by PKA-C^{WT} and PKA-C^{F100A}. The K_M and V_{max} values were obtained from a nonlinear least squares analysis of the concentration-dependent initial phosphorylation rates using a standard coupled enzyme activity. Error in k_{cat}/K_M was propagated from the error in K_M and k_{cat}.

	**PKA-C^{WT}**	**PKA-C^{F100A}**
V_{max}	0.322 ± 0.005	0.379 ± 0.009
K_M	30 ± 1	42 ±3
k_{cat}	15 ± 1	17 ± 1
k_{cat}/K_M	0.50 ± 0.04	0.41 ± 0.08

bioRxiv preprint doi: https://doi.org/10.1101/2023.09.12.557419; this version posted September 15, 2023. The copyright holder for this preprint (which was not certified by peer review) is the author/funder, who has granted bioRxiv a license to display the preprint in perpetuity. It is made available under aCC-BY 4.0 International license.

Supplementary Table 3. Changes in enthalpy, entropy, free energy, and dissociation constant for the binding of nucleotide to PKA-C^{WT} and PKA-C^{F100A}. All errors were calculated using triplicate measurements. Values for PKA-C^{WT} are re-printed for clarity but were originally published in Walker *et al.* [15]

	K_d (μM)	ΔG (kcal/mol)	ΔH (kcal/mol)	-TΔS (kcal/mol)	σ
PKA-C^{WT}	83 ± 8	-5.61 ± 0.06	-3.6 ± 0.1	-2.0 ± 0.1	N/A
PKA-C^{F100A}	73 ± 2	-5.7 ± 0.2	-21 ± 5	7 ± 3	N/A

bioRxiv preprint doi: https://doi.org/10.1101/2023.09.12.557419; this version posted September 15, 2023. The copyright holder for this preprint (which was not certified by peer review) is the author/funder, who has granted bioRxiv a license to display the preprint in perpetuity. It is made available under aCC-BY 4.0 International license.

Supplementary Table 4. Changes in enthalpy, entropy, free energy, and dissociation constant for the binding of PKI_{5-24} to apo and nucleotide-saturated $PKA-C^{WT}$ and $PKA-C^{F100A}$. All errors were calculated using triplicate measurements. The error in σ was propagated from the error in K_d. Values for $PKA-C^{WT}$ are re-printed for clarity but were originally published in Walker *et al.* [15].

PKI_{5-24} to apo kinase					
	K_d (μM)	ΔG (kcal/mol)	ΔH (kcal/mol)	-TΔS (kcal/mol)	σ
$PKA-C^{WT}$	17 ± 2	-6.57 ± 0.08	-10.8 ± 0.5	4.2 ± 0.5	N/A
$PKA-C^{F100A}$	5 ± 1	-7.7 ± 0.1	-17 ± 5	7 ± 3	N/A

PKI_{5-24} to ATPγN-saturated kinase					
	K_d (μM)	ΔG (kcal/mol)	ΔH (kcal/mol)	-TΔS (kcal/mol)	σ
$PKA-C^{WT}$	0.16 ± 0.02	-9.33 ± 0.07	-13.9 ± 0.5	4.6 ± 0.4	106 ± 18
$PKA-C^{F100A}$	2 ± 1	-7.9 ± 0.3	-17 ± 1	9 ± 1	3 ± 1
